# Supplementary figures and images for: The impact of different negative training data on regulatory sequence predictions
Source: PLoS One. 2020 Dec 1;15(12):e0237412. doi: 10.1371/journal.pone.0237412 (PMC7707526; doi:10.1371/journal.pone.0237412)

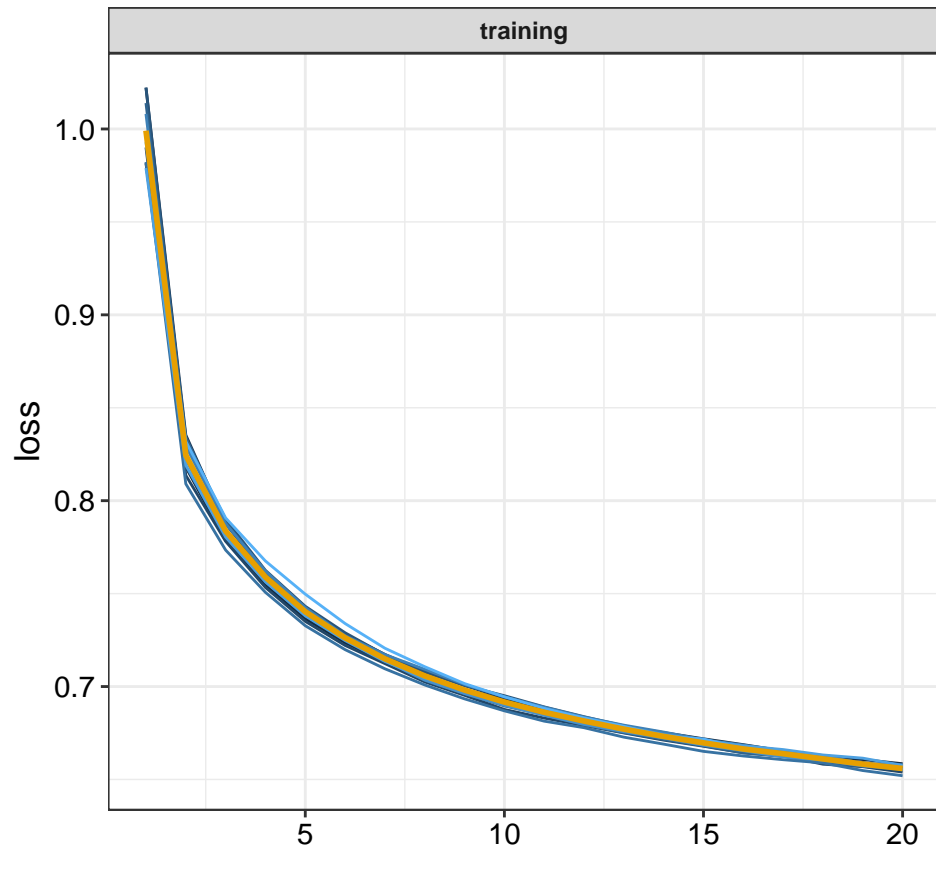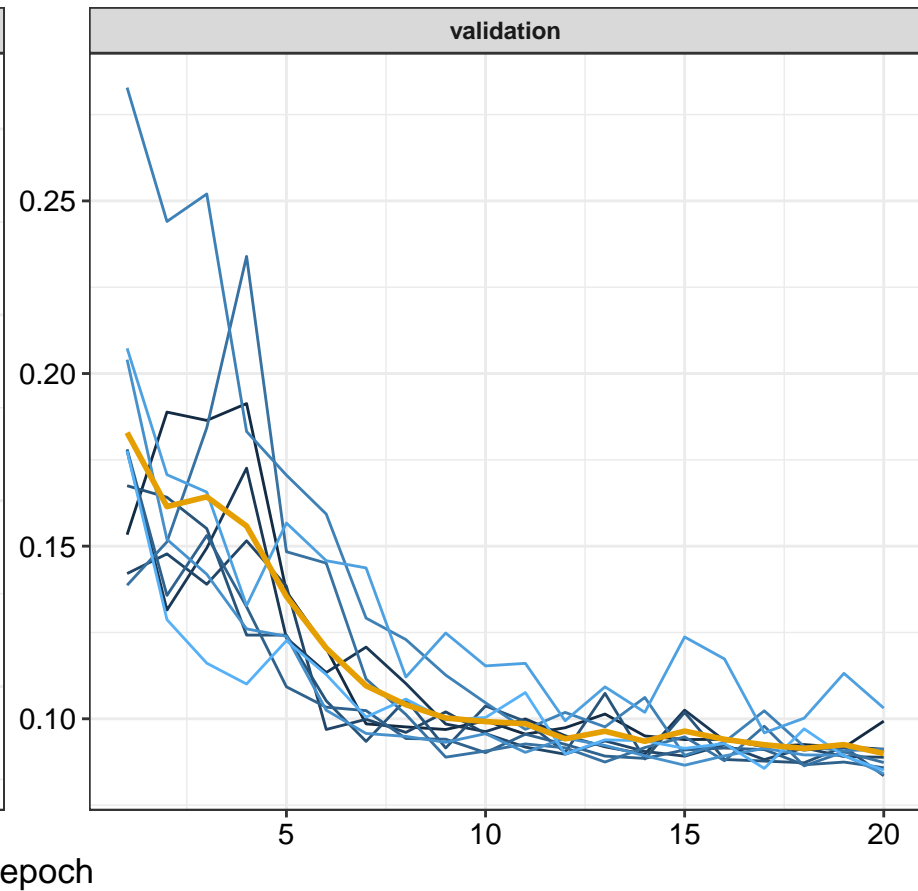

Supplement: S1 Fig — Each model was trained on a HeLa-S3 DHS (positive) training dataset and a 2-mer shuffled (negative) training dataset using the 2conv2norm classifier. Training was repeated 10 times and results are represented in different shades of blue while the mean values are represented in orange. Estimated loss in the training set and the validation set are displayed on the left and right, respectively. (PDF) [file pone.0237412.s001.pdf]

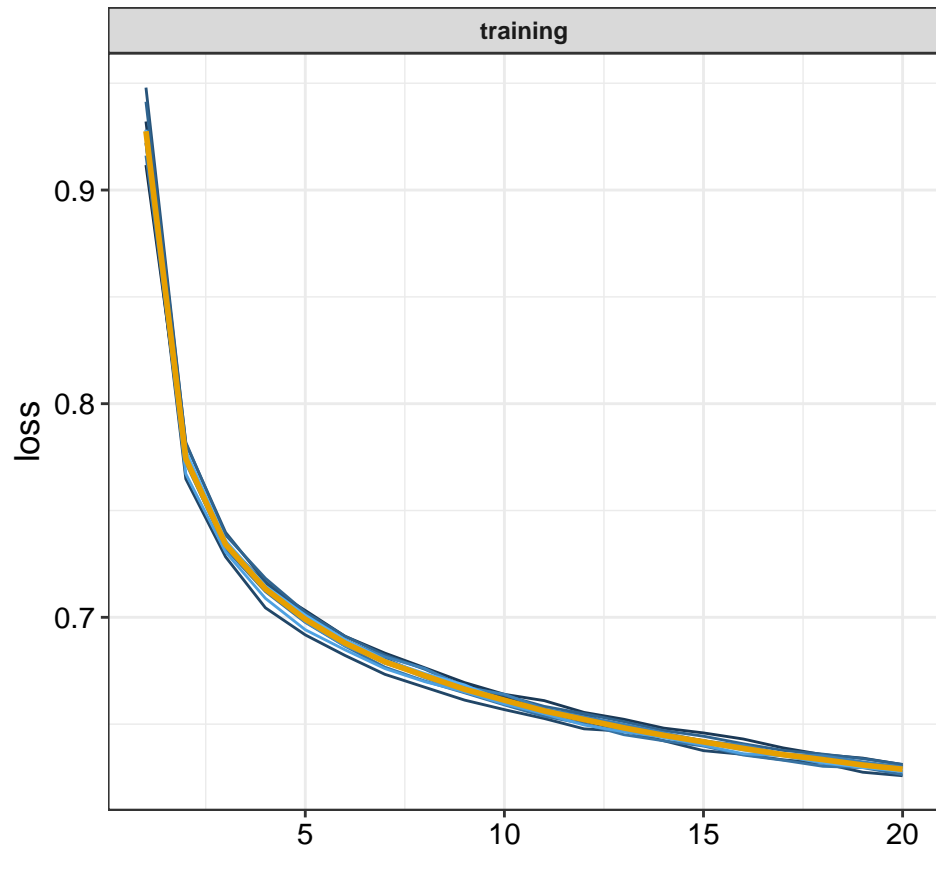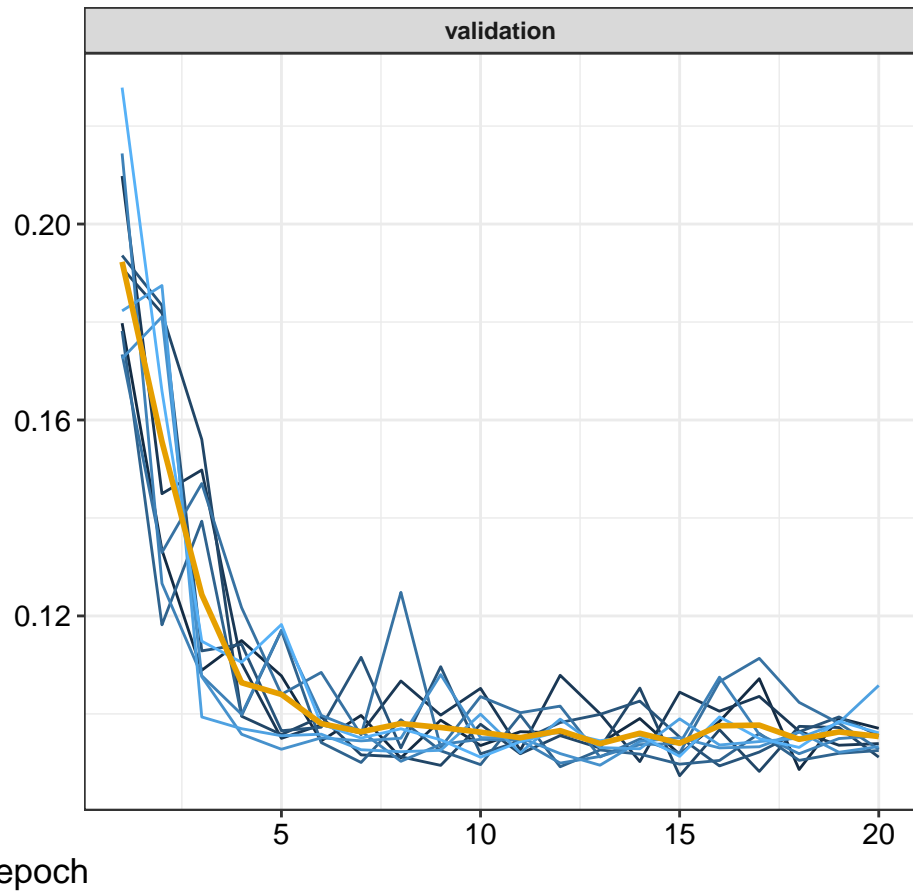

Supplement: S2 Fig — Each model was trained on a HeLa-S3 DHS (positive) training dataset and a 2-mer shuffled (negative) training dataset using the 4conv2pool4norm classifier. Training was repeated 10 times and results are represented in different shades of blue while the mean values are represented in orange. Estimated loss in the training set and the validation set are displayed on the left and right, respectively. (PDF) [file pone.0237412.s002.pdf]

DHS prediction on validation set

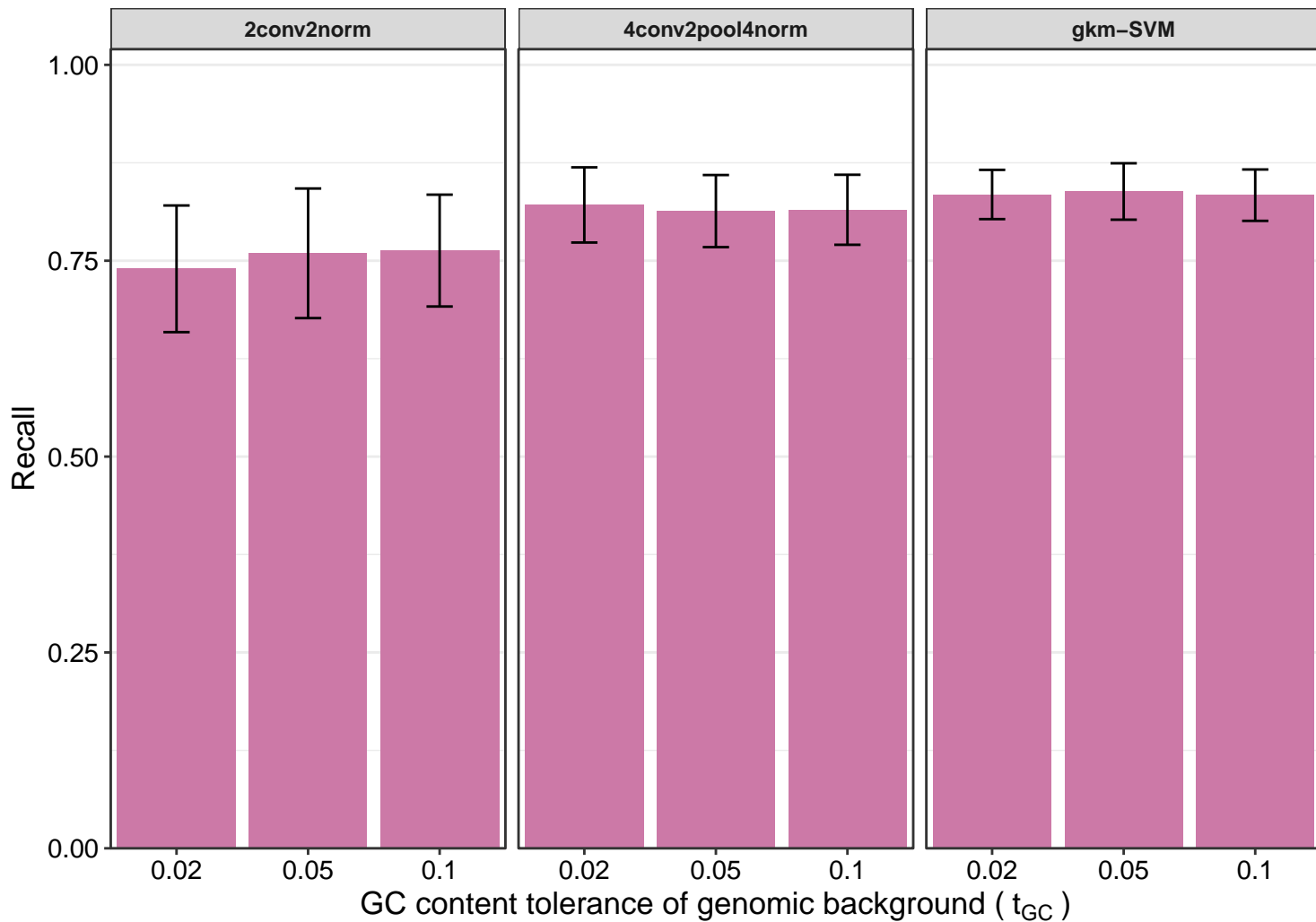

Supplement: S3 Fig — Each model was trained on a DHS (positive) training dataset and a genomic background (negative) training dataset and tested on a chromosome 21 hold-out validation set. Recall was calculated as a measure of model performance. For each classifier three different negative training sets are compared where the tolerances of differences in GC content composition (tGC) is varied. Each model was trained on data derived from one cell line. Bars represent the mean of multiple cell lines and technical replicates (n = 7 for gkm-SVM, n = 70 for CNNs: 10 replicates per cell line) while error bars represent the standard deviation. (PDF) [file pone.0237412.s003.pdf]

DHS prediction on validation set

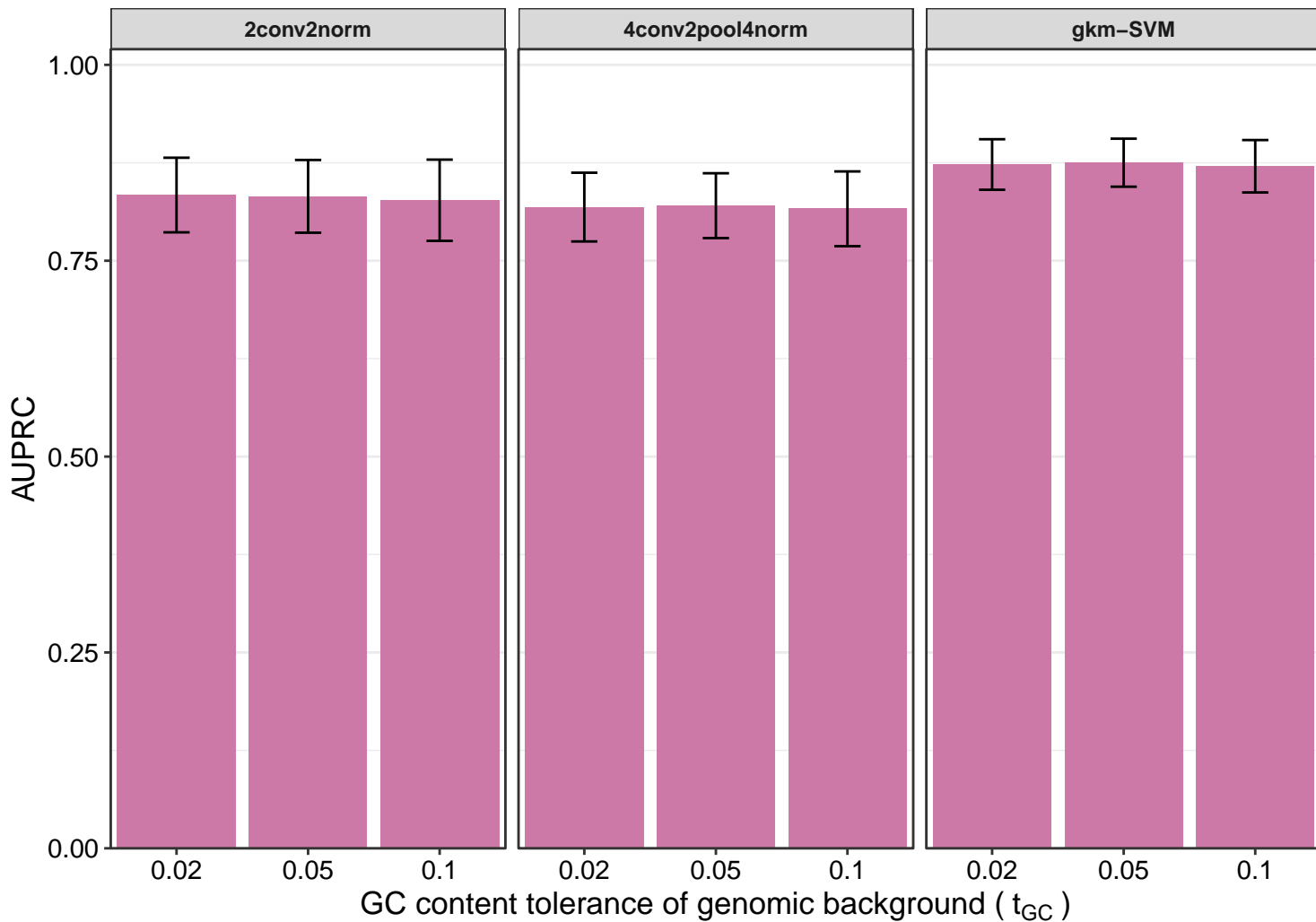

Supplement: S4 Fig — Each model was trained on a DHS (positive) training dataset and a genomic background (negative) training dataset and tested on a chromosome 21 hold-out validation set. Area under precision recall curve (AUPRC) was calculated as a measure of model performance. For each classifier three different negative training sets are compared where the tolerances of differences in GC content composition (tGC) is varied. Each model was trained on data derived from one cell line. Bars represent the mean of multiple cell lines and technical replicates (n = 7 for gkm-SVM, n = 70 for CNNs: 10 replicates per cell line) while error bars represent the standard deviation. (PDF) [file pone.0237412.s004.pdf]

DHS prediction on validation set

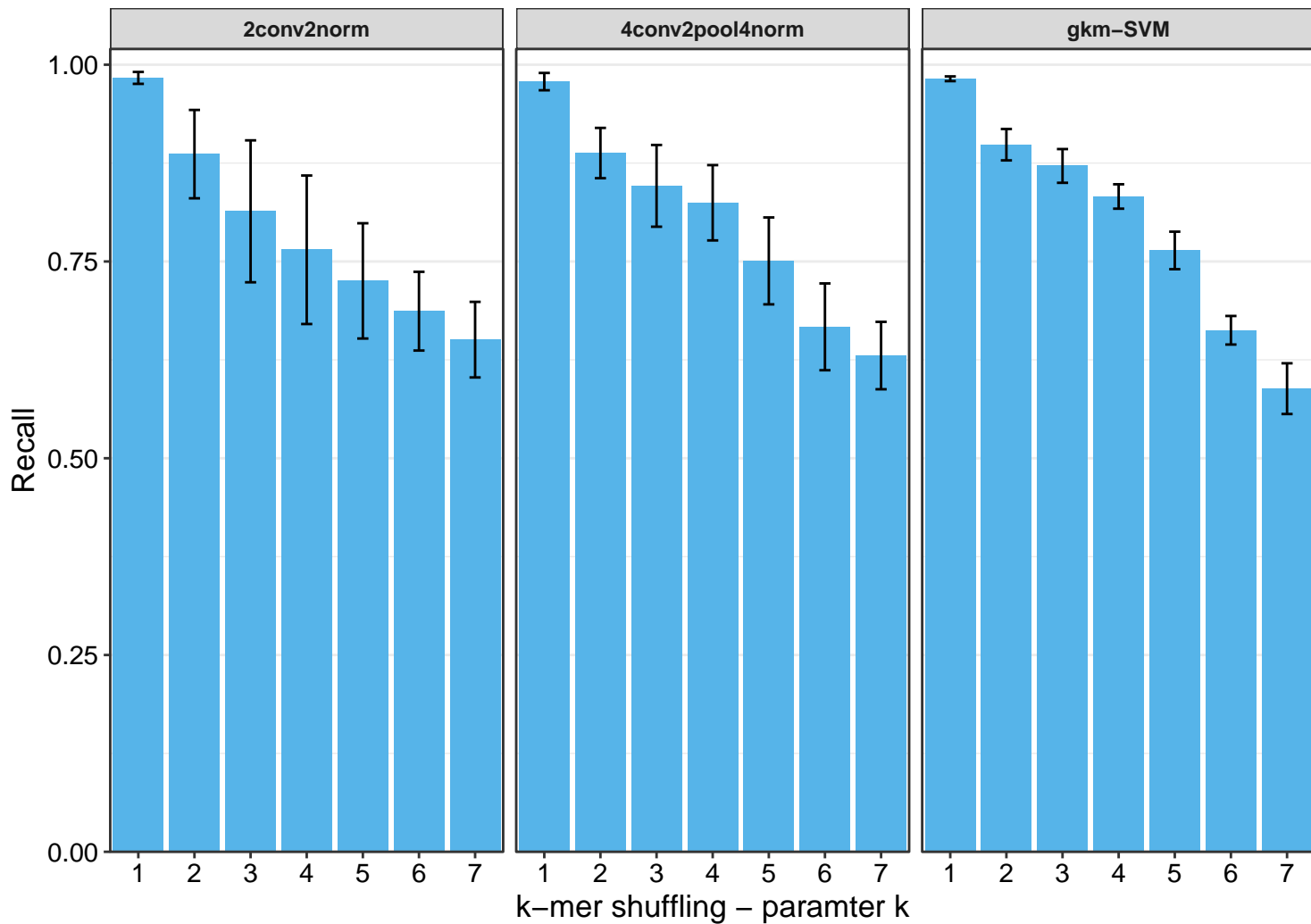

Supplement: S5 Fig — Each model was trained on a DHS (positive) training dataset and a k-mer shuffled (negative) training dataset and tested on a chromosome 21 hold-out validation set. Recall was calculated as a measure of model performance. For each classifier seven different negative training sets are compared where the size of preserved k-mers during shuffling is varied. Each model was trained on data derived from one cell line. Bars represent the mean of multiple cell lines and technical replicates (n = 7 for gkm-SVM, n = 70 for CNNs: 10 replicates per cell line) while error bars represent the standard deviation. (PDF) [file pone.0237412.s005.pdf]

DHS prediction on validation set

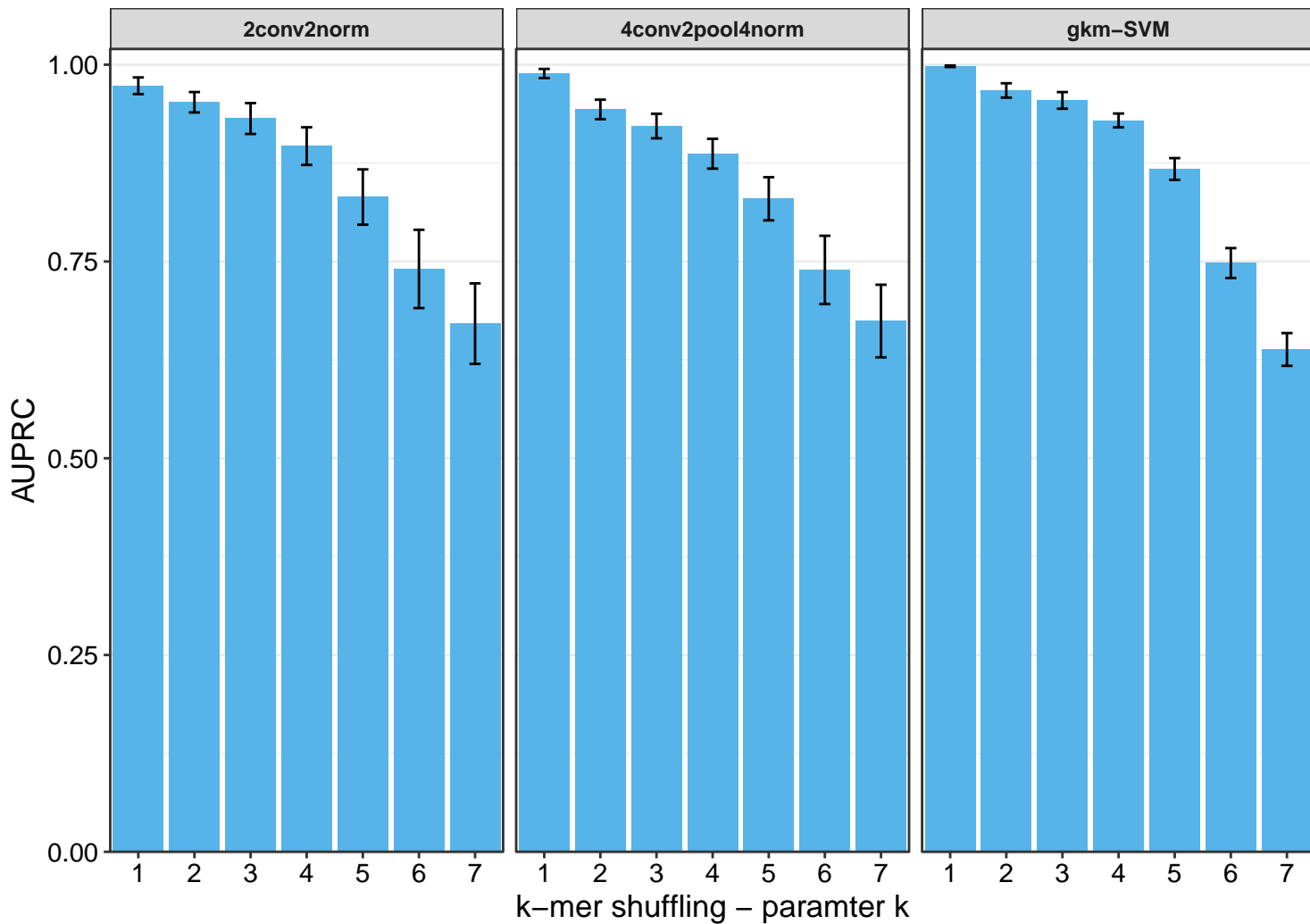

Supplement: S6 Fig — Each model was trained on a DHS (positive) training dataset and a k-mer shuffled (negative) training dataset and tested on a chromosome 21 hold-out validation set. Area under precision recall curve (AUPRC) was calculated as a measure of model performance. For each classifier seven different negative training sets are compared where the size of preserved k-mers during shuffling is varied. Each model was trained on data derived from one cell line. Bars represent the mean of multiple cell lines and technical replicates (n = 7 for gkm-SVM, n = 70 for CNNs: 10 replicates per cell line) while error bars represent the standard deviation. (PDF) [file pone.0237412.s006.pdf]

# TFBS motifs in training sequences

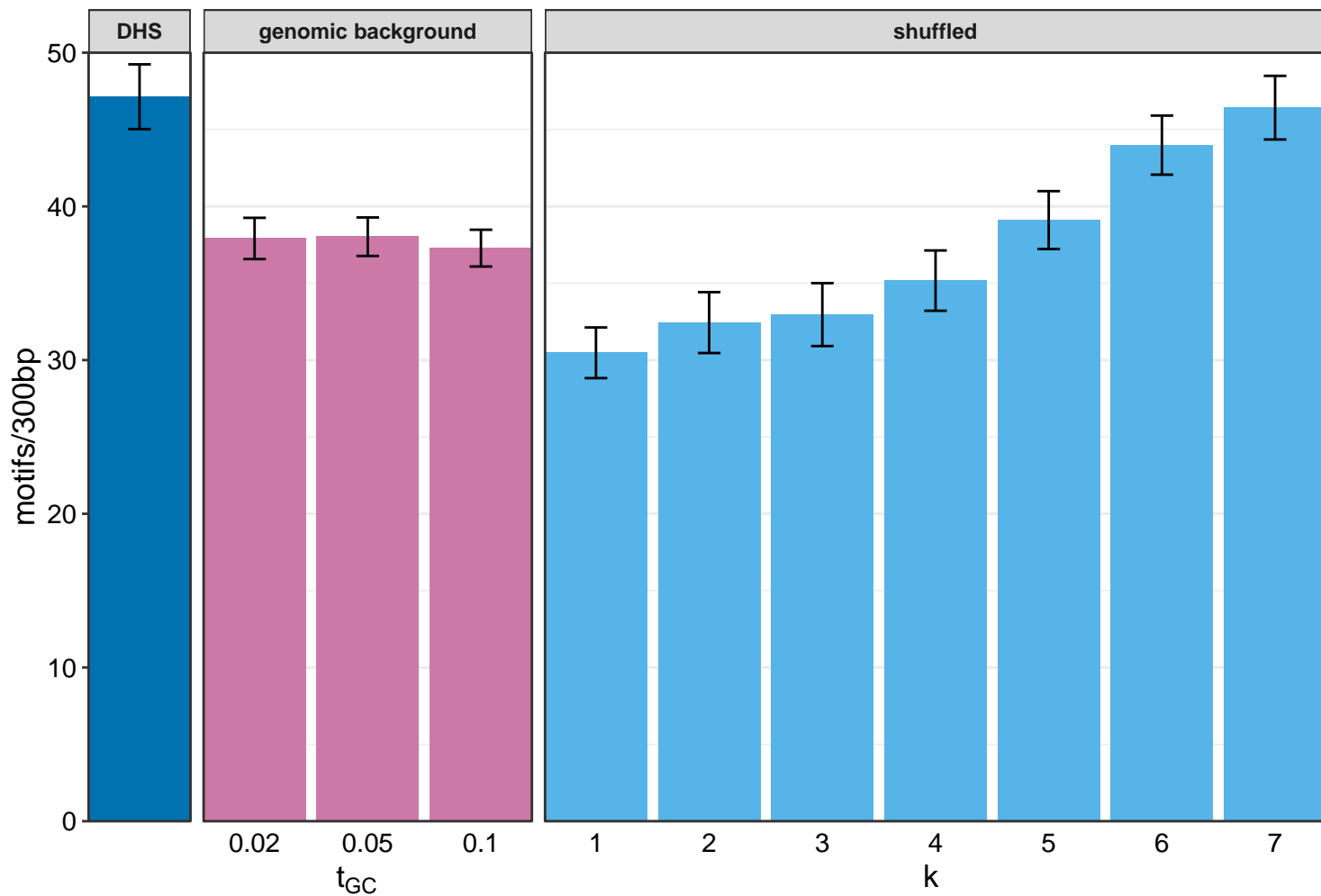

Supplement: S7 Fig — Known human transcription factor binding site (TFBS) motifs were matched in training sequences of different datasets from different cell lines (n = 7). Bars represent the mean value, error bars the standard deviation. (PDF) [file pone.0237412.s007.pdf]

## DHS prediction on test set

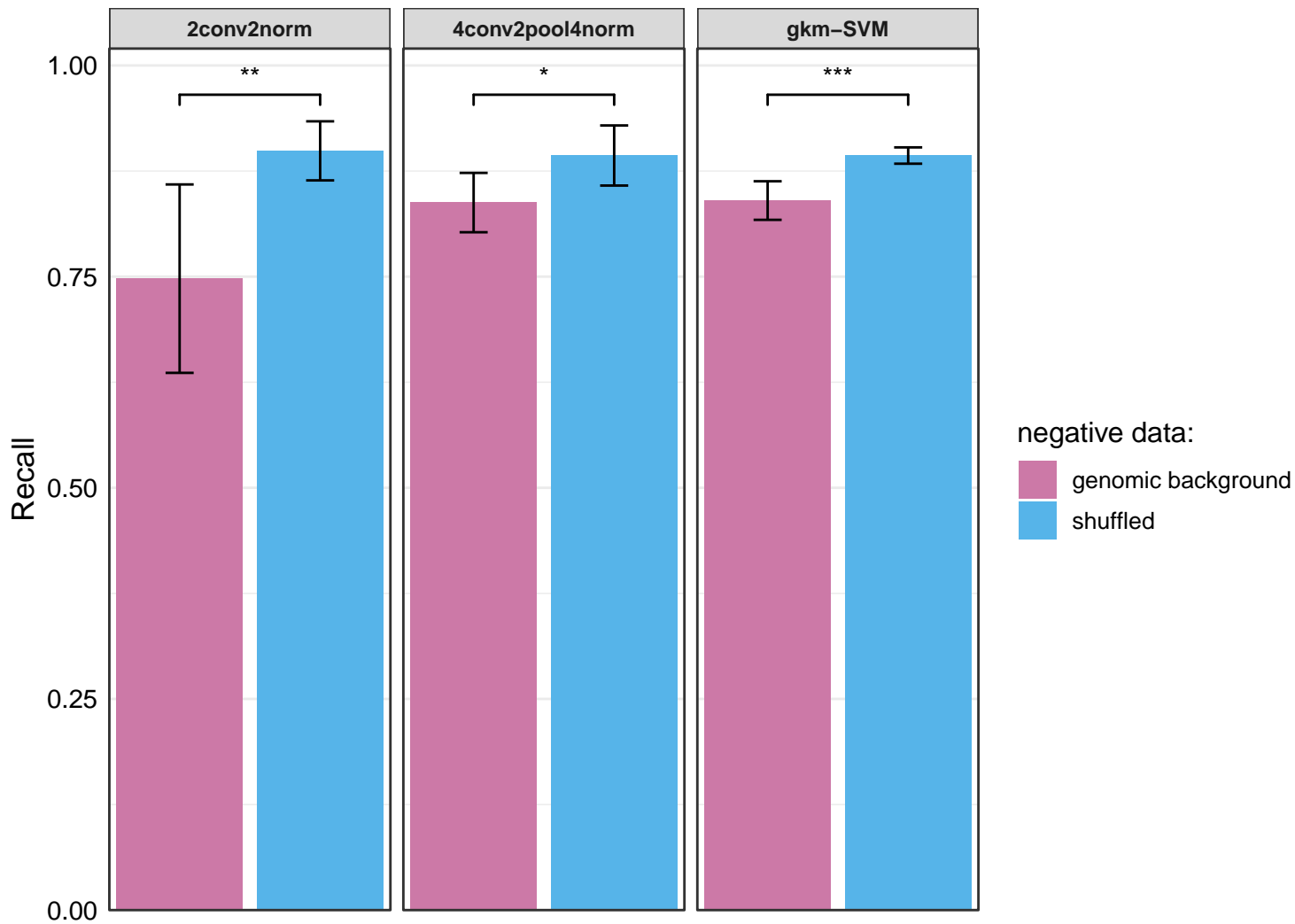

Supplement: S8 Fig — Models were trained on sequences of DHS regions (positive) with corresponding sets of negative sequences and tested on a chromosome 8 hold-out test set. For each classifier two different negative training sets are compared; sequences were either chosen from genomic background (tGC = 0.02) or generated by shuffling positive sequences and preserving k-mer counts (k = 2). Recall was calculated to compare model performance. Seven models were trained on data derived for specific cell lines, bars represent the mean and error bars the standard deviations across models. Pairwise comparisons were performed with Wilcoxon signed-rank tests and asterisks represent significance levels (*p<0.05, **p<0.01, ***p<0.001). (PDF) [file pone.0237412.s008.pdf]

## DHS prediction on test set

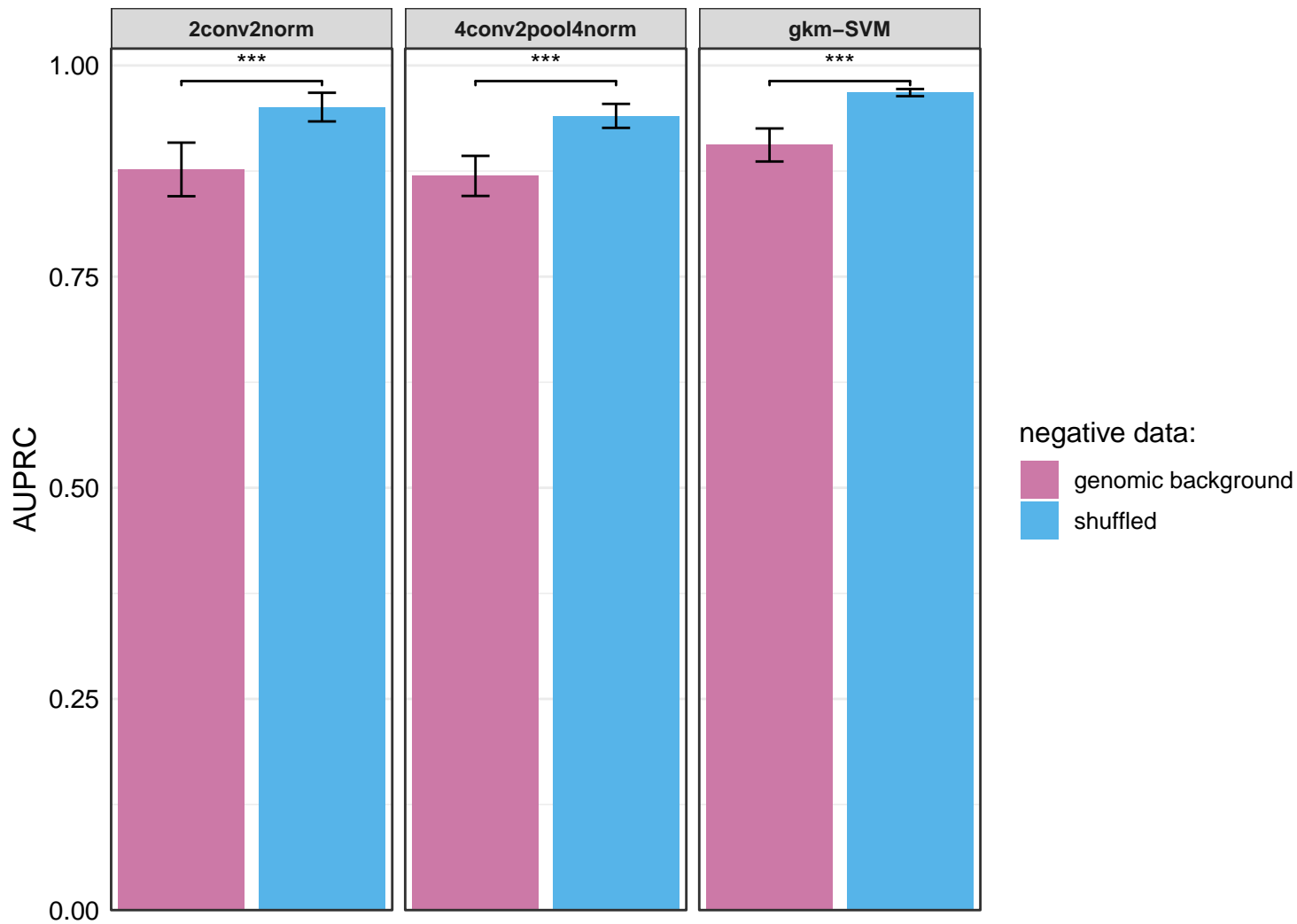

Supplement: S9 Fig — Each model was trained on a DHS (positive) training dataset and a set of neutral sequences (negative) and tested on a chromosome 8 hold-out test set. Recall was calculated as a measure of model performance. For each classifier two different negative training sets are compared. Sequences were either chosen from genomic background (tGC = 0.02) or generated by shuffling positive sequences and preserving k-mer counts (k = 2). Each model was trained on data derived from one cell line. Bars represent the mean of multiple cell lines (n = 7) while error bars represent standard deviations. Pairwise comparisons were performed with Wilcoxon signed-rank test and asterisks represent significance levels (*p<0.05, **p<0.01, ***p<0.001). (PDF) [file pone.0237412.s009.pdf]

**A**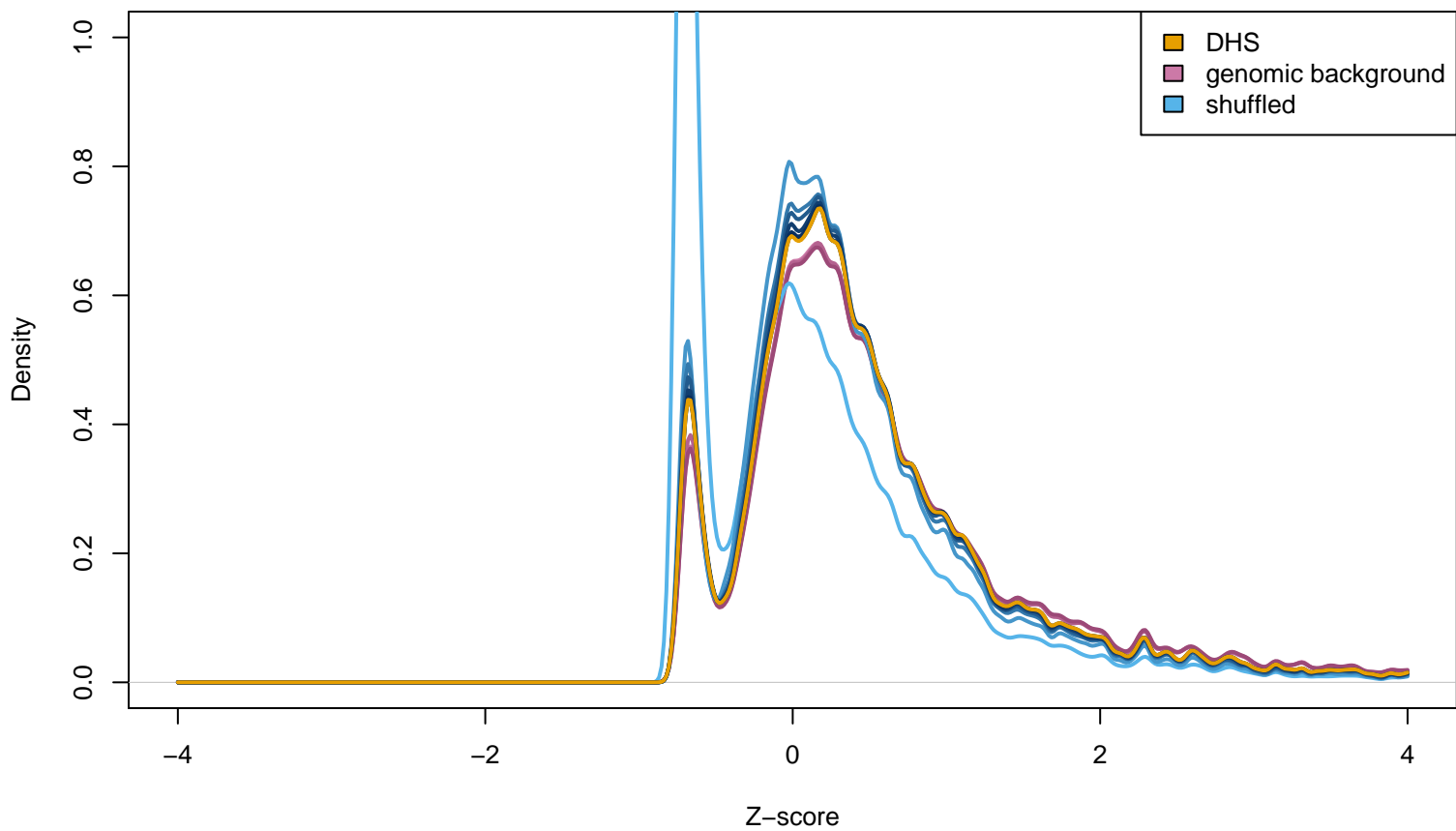**B****2conv2norm**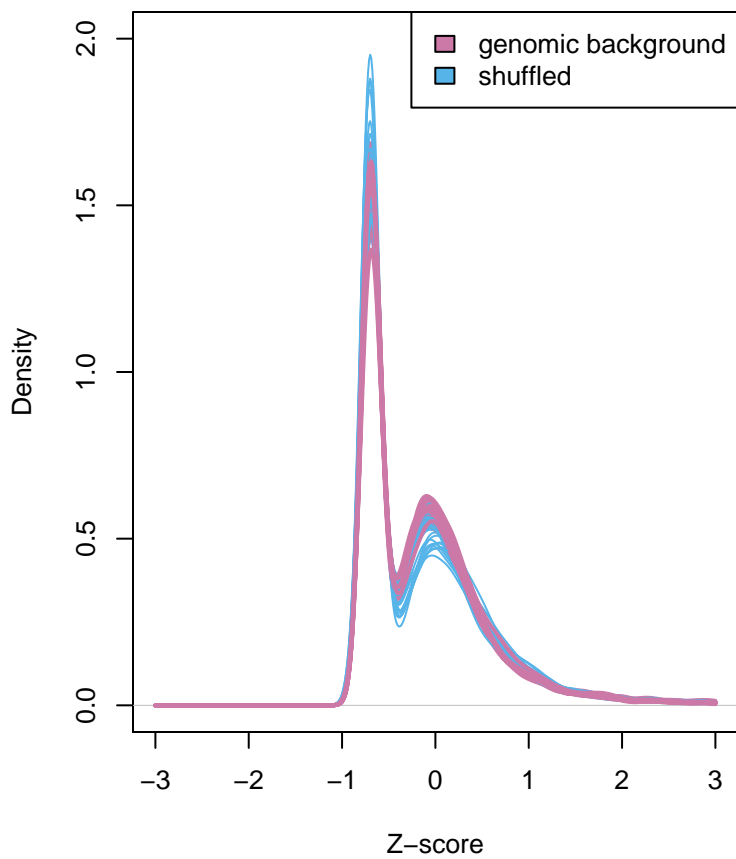**C****4conv2pool4norm**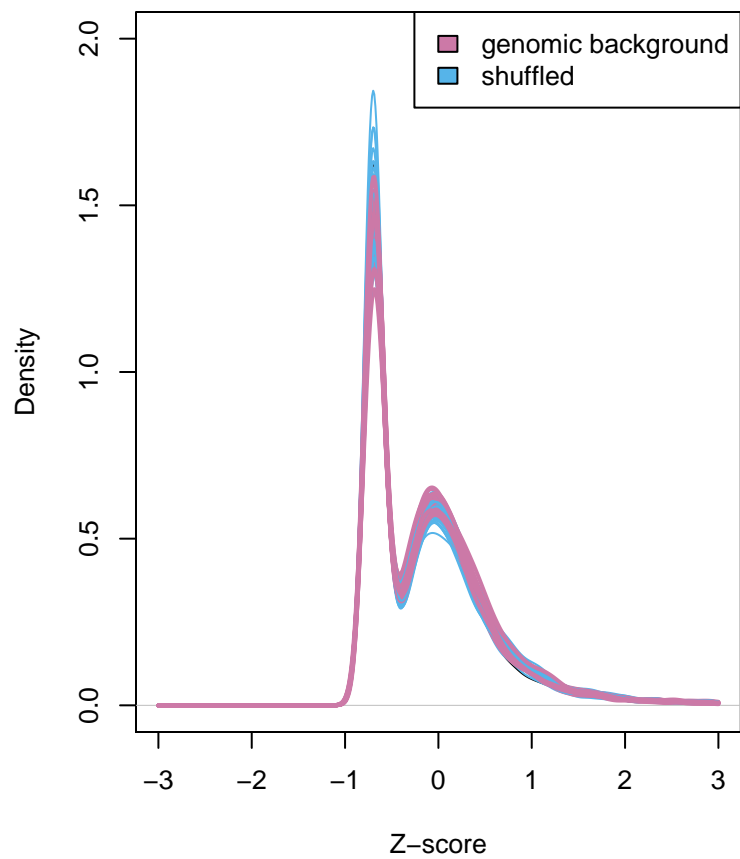

Supplement: S10 Fig — Exemplary for all cell-types, the figure shows results for HeLa-S3. Genomic frequency of 8-mers was extracted across all major human chromosomes and Z-Score transformed (i.e. mean-centered and standard deviation normalized to one). Panel (A) shows the genomic frequency of 8-mers in the test sets split out as DHS sites (orange, positive class), negative genomic background sequences (shades of red, from low to high) and different negative k-mer shuffles (shades of blue, from low to high). Smaller k-mer shuffles contain more rare genomic 8-mers. Panel (B) shows the distribution of the genomic 8-mer frequency for the top 100 sequences for each of 128 kernels in the first convolutional layer for 2conv2norm (left) and 4conv2pool4norm (right) architectures. (PDF) [file pone.0237412.s010.pdf]

A

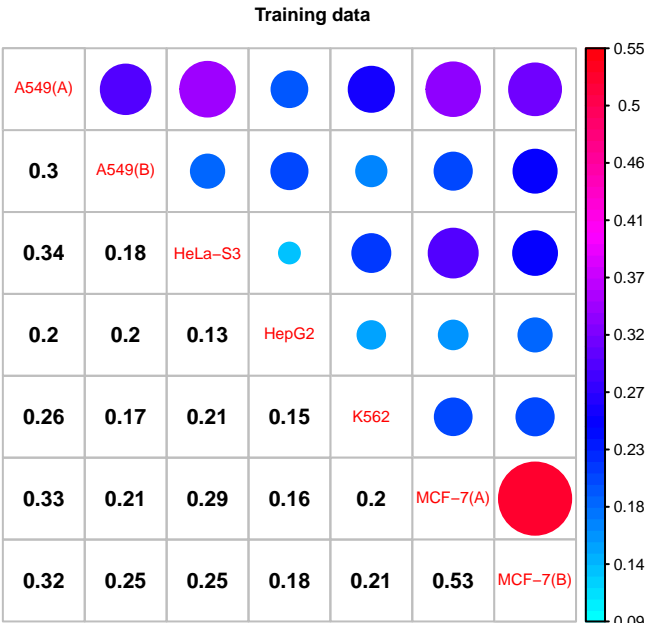

B

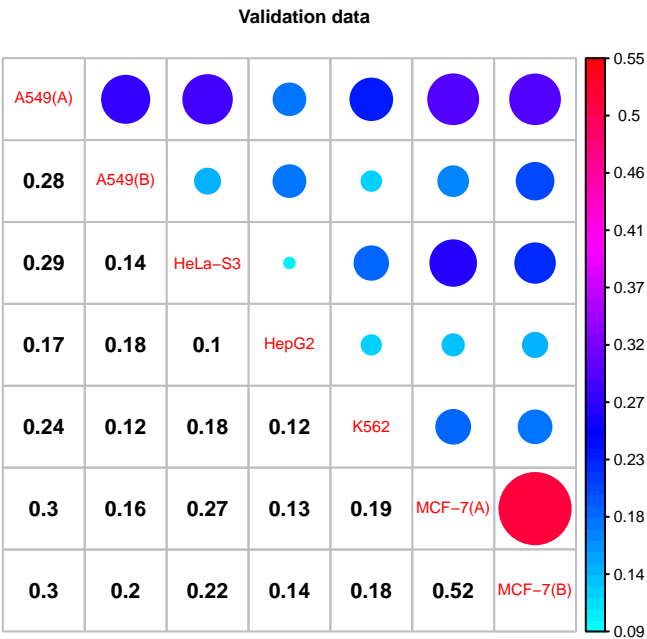

C

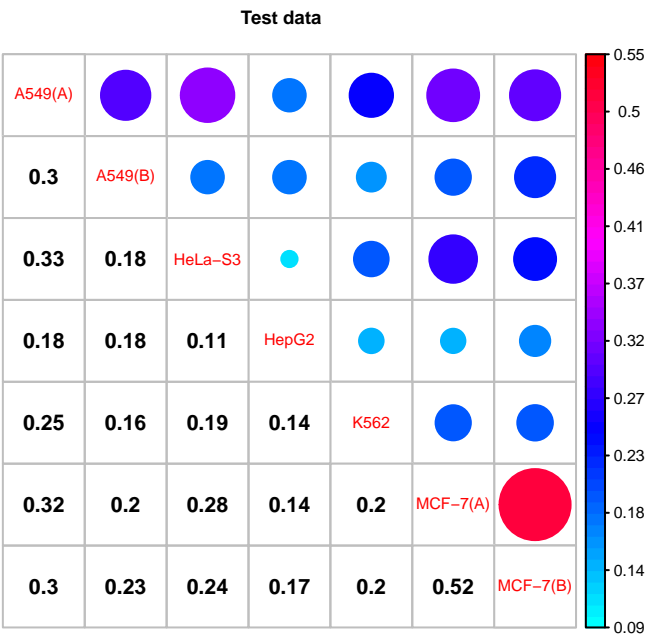

Supplement: S11 Fig — Pairwise sequence overlap in (a) training, (b) validation and (c) test sets. We determined the overlap of merged peak sets across experiments in the same cell-type and across cell-types. For peaks to be considered overlapping between datasets, we required a 70% overlap in their coordinate ranges. We calculated pairwise overlap as number of overlapping peaks divided by the number of peaks in the union of both data sets. Datasets are named according to S1 Table. (PDF) [file pone.0237412.s011.pdf]

# Liver enhancer activity prediction

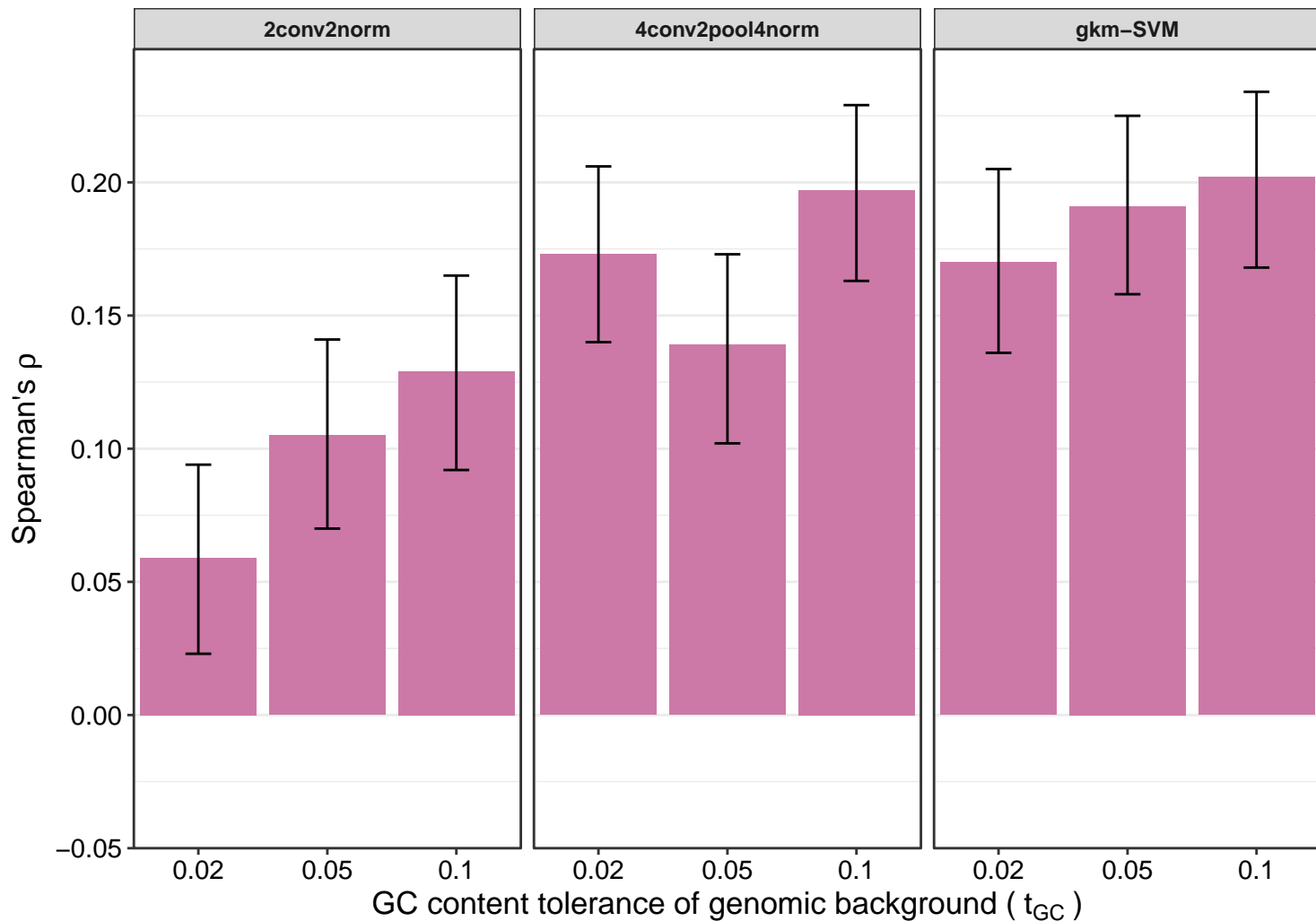

Supplement: S14 Fig — Models were trained on HepG2 DHS sequences (positive) and genomic background sequences (negative), where different genomic background sets result from a variation of the GC content tolerance (tGC). Models were tested on enhancer activity readouts in HepG2 cells [25]. Spearman rank correlation of predicted scores and log2 RNA/DNA ratios was used to evaluate model performance. Error bars represent 95% confidence intervals. (PDF) [file pone.0237412.s014.pdf]

# Liver enhancer activity prediction

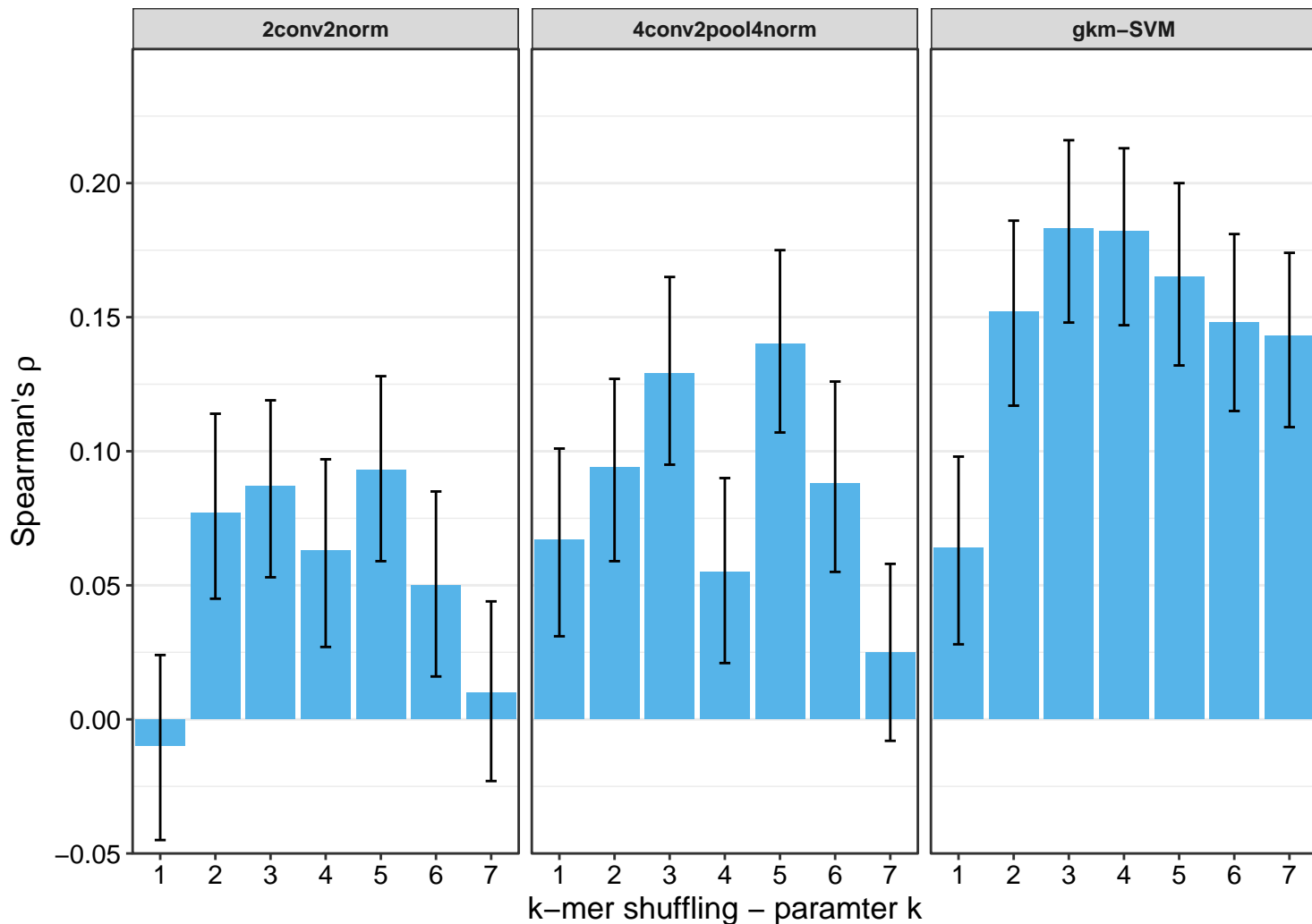

Supplement: S15 Fig — Models were trained on HepG2 DHS sequences (positive) and genomic background sequences (negative), where different genomic background sets result from a variation of the size of preserved k-mers. Models were tested on enhancer activity readouts in HepG2 cells [25]. Spearman rank correlation of predicted scores and log2 RNA/DNA ratios was used to evaluate model performance. Error bars represent 95% confidence intervals. (PDF) [file pone.0237412.s015.pdf]

# Liver enhancer activity prediction

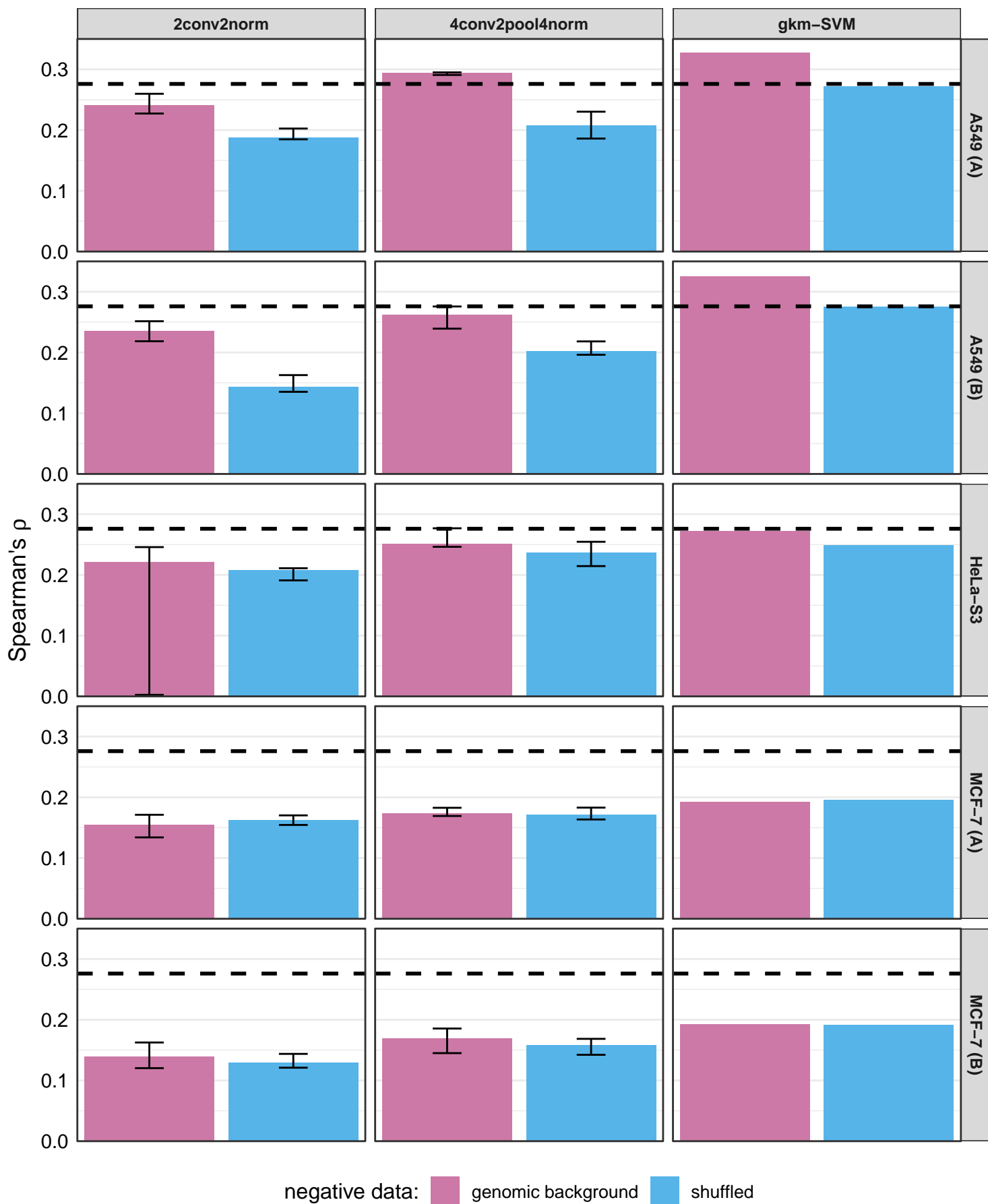

Supplement: S16 Fig — Models were trained either on DHS sequences active in A549, HeLa-S3 or MCF-7 cells (positive) and neutral sequences (negative), where different negative sets are composed of genomic background (tGC = 0.1) or shuffled (k = 3) sequences. Models were tested on activity readouts of enhancer sequences in HepG2 cells [25]. Spearman rank correlation of predicted scores and log2 RNA/DNA ratios was used to evaluate model performance. For 2conv2norm and 4conv2pool4norm bars represent the median of multiple replicates (n = 10) while error bars represent 1st and 3rd quartiles. The dashed black line represents a reference value (Spearman’s ρ = 0.276) achieved previously [25]. (PDF) [file pone.0237412.s016.pdf]

**GC content distributions in training data**

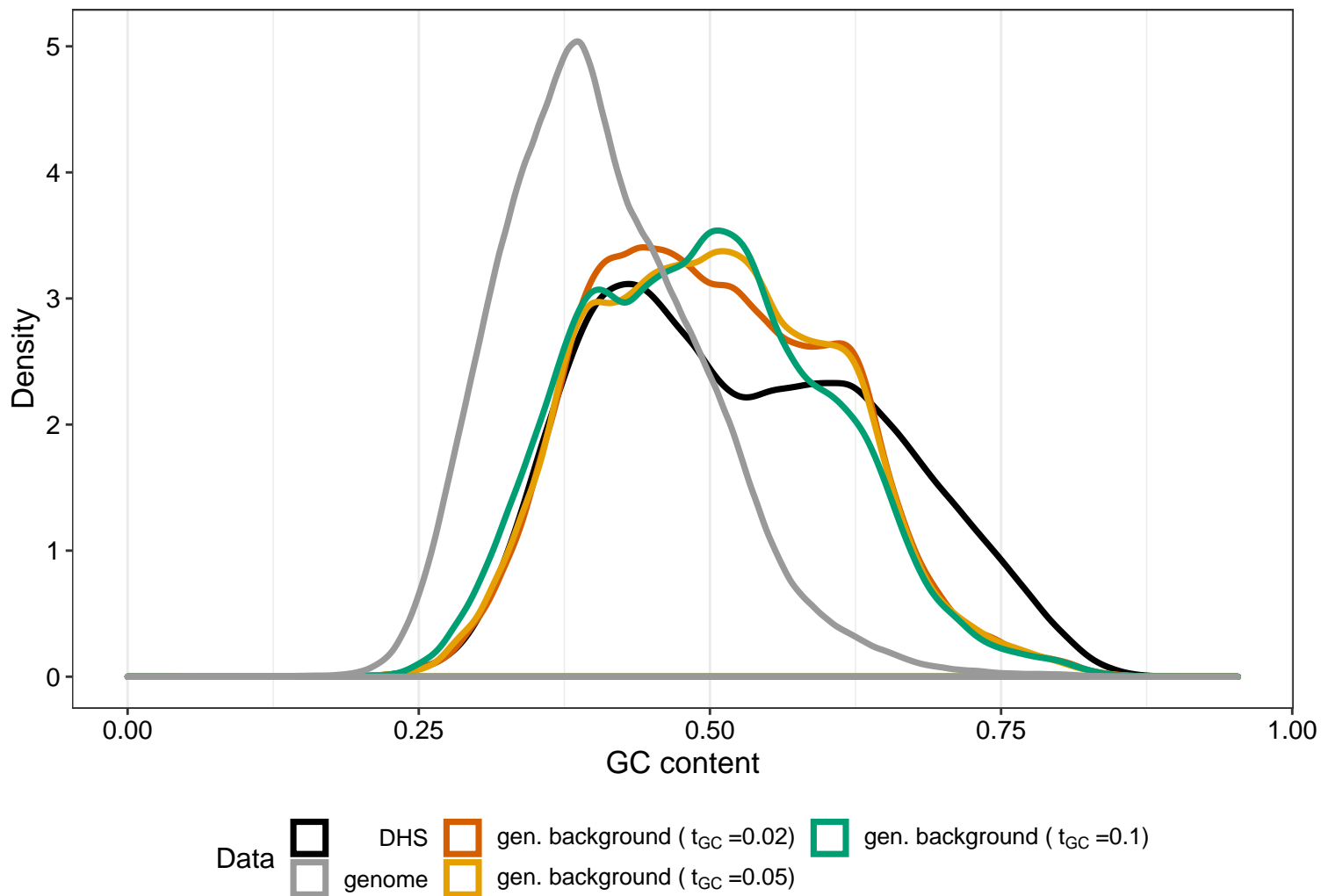

Supplement: S17 Fig — The distribution of the sequences’ GC contents in a dataset of active DHS regions in HepG2, three corresponding genomic background datasets with varied GC content tolerance (tGC) and a set of random 300 bp sequences from the genome is shown. (PDF) [file pone.0237412.s017.pdf]
