# Supplementary material for: The impact of different negative training data on regulatory sequence predictions
Source: PLoS One. 2020 Dec 1;15(12):e0237412. doi: 10.1371/journal.pone.0237412 (PMC7707526; doi:10.1371/journal.pone.0237412)
Supplement: S12 Fig — Models were trained on DHS sequences (positive) active in HeLa-S3 cells and neutral sequences from genomic background (negative) with varied GC content tolerance (tGC). Models were tested on DHS sequences specifically active in HeLa-S3 (positive) and DHS sequences active only in one or multiple other cell lines (A549, HepG2, K562, MCF-7) (negative). (A) and (B) show ROC and PR curves for 2conv2norm models, (C) and (D) show ROC and PR curves for 4conv2pool4norm models, (E) and (F) show ROC and PR curves for gkm-SVM models. Corresponding AUROC and AUPRC values are included. (PDF) [file pone.0237412.s012.pdf]

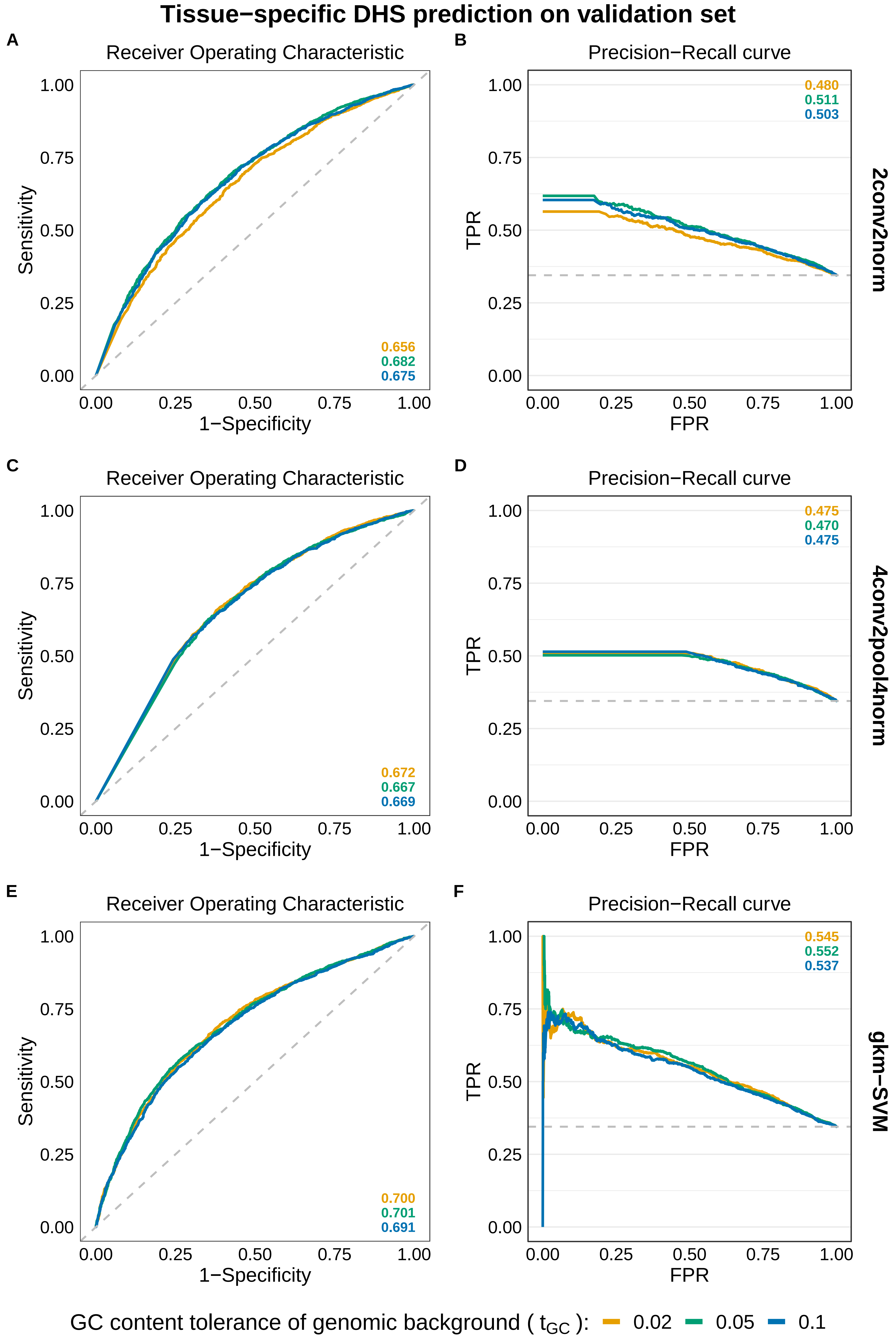

Tissue−specific DHS prediction on validation set
A
Receiver Operating Characteristic
Sensitivity
1−Specificity
0.656
0.682
0.675
B
Precision−Recall curve
TPR
FPR
0.480
0.511
0.503
2conv2norm
C
Receiver Operating Characteristic
Sensitivity
1−Specificity
0.672
0.667
0.669
D
Precision−Recall curve
TPR
FPR
0.475
0.470
0.475
4conv2pool4norm
E
Receiver Operating Characteristic
Sensitivity
1−Specificity
0.700
0.701
0.691
F
Precision−Recall curve
TPR
FPR
0.545
0.552
0.537
gkm−SVM
GC content tolerance of genomic background (t_{GC}): 0.02 0.05 0.1
