# Supplementary material for: The impact of different negative training data on regulatory sequence predictions
Source: PLoS One. 2020 Dec 1;15(12):e0237412. doi: 10.1371/journal.pone.0237412 (PMC7707526; doi:10.1371/journal.pone.0237412)
Supplement: S13 Fig — Models were trained on DHS sequences (positive) active in HeLa-S3 cells and neutral sequences from genomic background (negative) with varied size of preserved k-mers. Models were tested on DHS sequences specifically active in HeLa-S3 (positive) and DHS sequences active only in one or multiple other cell lines (A549, HepG2, K562, MCF-7) (negative). (A) and (B) show ROC and PR curves for 2conv2norm models, (C) and (D) show ROC and PR curves for 4conv2pool4norm models, (E) and (F) show ROC and PR curves for gkm-SVM models. Corresponding AUROC and AUPRC values are included. (PDF) [file pone.0237412.s013.pdf]

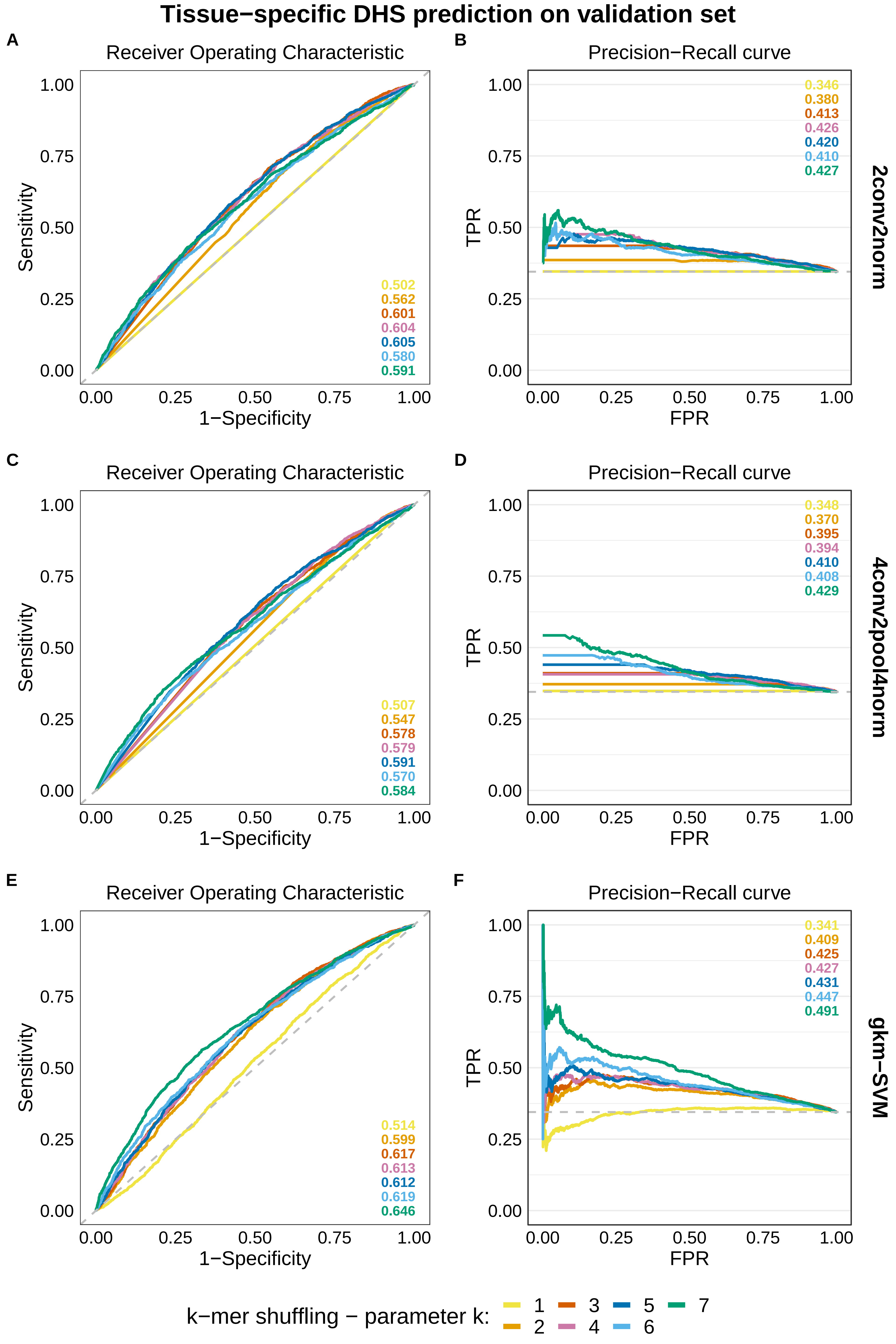

Tissue−specific DHS prediction on validation set
A
Receiver Operating Characteristic
Sensitivity
1−Specificity
0.502
0.562
0.601
0.604
0.605
0.580
0.591
B
Precision−Recall curve
TPR
FPR
0.346
0.380
0.413
0.426
0.420
0.410
0.427
2conv2norm
C
Receiver Operating Characteristic
Sensitivity
1−Specificity
0.507
0.547
0.578
0.579
0.591
0.570
0.584
D
Precision−Recall curve
TPR
FPR
0.348
0.370
0.395
0.394
0.410
0.408
0.429
4conv2pool4norm
E
Receiver Operating Characteristic
Sensitivity
1−Specificity
0.514
0.599
0.617
0.613
0.612
0.619
0.646
F
Precision−Recall curve
TPR
FPR
0.341
0.409
0.425
0.427
0.431
0.447
0.491
gkm−SVM
k−mer shuffling − parameter k: 1 2 3 4 5 6 7
