# Supplementary material for: The impact of different negative training data on regulatory sequence predictions
Source: PLoS One. 2020 Dec 1;15(12):e0237412. doi: 10.1371/journal.pone.0237412 (PMC7707526; doi:10.1371/journal.pone.0237412)
Supplement: S1 Table — The number of DHS sequences is given after merging replicates and exclusion of alternative haplotypes, unlocalized genomic contigs and sequences containing non-ATCG bases. The datasets were split up into training, validation (chromosome 21) and test (chromosome 8) sets. The number of samples in these sets are given in the respective columns. Experiment and Replicate IDs are referring to ENCODE accessions. (PDF) [file pone.0237412.s018.pdf]

S1 Table: Overview of DNase-seq datasets. The number of DHS sequences is given after merging replicates and exclusion of alternative haplotypes, unlocalized genomic contigs and sequences containing non-ATCG bases. The datasets were split up into training, validation (chromosome 21) and test (chromosome 8) sets. The number of samples in these sets are given in the respective columns. Experiment and Replicate IDs are referring to ENCODE accessions.

Cell line	Experiment ID	Replicate ID	# training	# validation	# test
A549 (A)	ENCSR000ELW	ENCFF529HMB,<br>ENCFF823UOG	192467	1950	9744
A549 (B)	ENCSR136DNA	ENCFF045PYX,<br>ENCFF079DJV,<br>ENCFF135JRM,<br>ENCFF698UAH	85880	759	4510
HeLa-S3	ENCSR959ZXU	ENCFF950NDW,<br>ENCFF736UEX	280678	3557	14643
HepG2	ENCSR000ENP	ENCFF470WDP,<br>ENCFF630RQY	122510	1445	5480
K562	ENCSR000EOY	ENCFF821KDJ,<br>ENCFF433TIR	222364	2970	10183
MCF-7 (A)	ENCSR000EPH	ENCFF961ZCT,<br>ENCFF229SJW	212282	2738	10973
MCF-7 (B)	ENCSR000EPJ	ENCFF751SAV,<br>ENCFF636FXP	140577	1830	7042
