# Supplementary material for: The impact of different negative training data on regulatory sequence predictions
Source: PLoS One. 2020 Dec 1;15(12):e0237412. doi: 10.1371/journal.pone.0237412 (PMC7707526; doi:10.1371/journal.pone.0237412)
Supplement: S2 Table — Tissue-specific positive samples are DHS sequences of one cell line not overlapping with DHS sequences of the other cell lines. In contrast, negative samples are DHS sequences of other cell lines not overlapping with the first cell line. For A549, one dataset was chosen (B, named according to S1 Table). For MCF-7 one dataset was chosen (B, named according to S1 Table). The number of DHS sequences is given after exclusion of alternative haplotypes, unlocalized genomic contigs and sequences containing non-ATCG bases. The validation and test sets contain sequences located on chromosome 21 and 8, respectively. (PDF) [file pone.0237412.s019.pdf]

S2 Table: Overview of tissue-specific validation and test sets. Tissue-specific positive samples are DHS sequences of one cell line not overlapping with DHS sequences of the other cell lines. In contrast, negative samples are DHS sequences of other cell lines not overlapping with the first cell line. For A549, one dataset was chosen (B, named according to S1 Table). For MCF-7 one dataset was chosen (B, named according to S1 Table). The number of DHS sequences is given after exclusion of alternative haplotypes, unlocalized genomic contigs and sequences containing non-ATCG bases. The validation and test sets contain sequences located on chromosome 21 and 8, respectively.

	Validation set		Test set	
Cell line	# positive	# negative	# positive	# negative
A549	48	7658	614	26725
HeLa-S3	1839	3493	7309	11998
HepG2	536	6893	1997	27118
K562	1524	4473	4761	19148
MCF-7	548	5766	1808	22340
