# Supplementary material for: The impact of different negative training data on regulatory sequence predictions
Source: PLoS One. 2020 Dec 1;15(12):e0237412. doi: 10.1371/journal.pone.0237412 (PMC7707526; doi:10.1371/journal.pone.0237412)
Supplement: S3 Table — The column named ‘Size’ provides the convolutional kernel size, the max-pooling window size, the relative dropout size and the dense layer size depending on information given in column ‘Layer type’. (PDF) [file pone.0237412.s020.pdf]

S3 Table: Layer properties of 4conv2pool4norm network. The column named 'Size' provides the convolutional kernel size, the max-pooling window size, the relative dropout size and the dense layer size depending on information given in column 'Layer type'.

Layer ID	Layer type	Activation function	Size	Output shape
0	Input	-	-	4x1x300
1	Conv	ReLU	128x4x1x8	128x1x293
2	Norm	-	-	128x1x293
3	Conv	ReLU	128x128x1x8	128x1x286
4	Norm	-	-	128x1x286
5	MaxPool	-	1x2	128x1x143
6	Conv	ReLU	64x128x1x3	64x1x141
7	Norm	-	-	64x1x141
8	Conv	ReLU	64x64x1x3	64x1x139
9	Norm	-	-	64x1x139
10	MaxPool	-	1x2	64x1x69
11	Flatten	-	-	4416
12	Dense	linear	256	256
13	Dropout	-	0.5	256
14	Dense	linear	128	128
15	Dense	softmax	2	2
