# Supplementary material for: The impact of different negative training data on regulatory sequence predictions
Source: PLoS One. 2020 Dec 1;15(12):e0237412. doi: 10.1371/journal.pone.0237412 (PMC7707526; doi:10.1371/journal.pone.0237412)
Supplement: S4 Table — The column named ‘Size’ provides the convolutional kernel size, the max-pooling window size, the relative dropout size and the dense layer size depending on information given in column ‘Layer type’. (PDF) [file pone.0237412.s021.pdf]

S4 Table: Layer properties of 2conv2norm network. The column named ‘Size’ provides the convolutional kernel size, the max-pooling window size, the relative dropout size and the dense layer size depending on information given in column ‘Layer type’.

Layer ID	Layer type	Activation function	Size	Output shape
0	Input	-	-	4x1x300
1	Conv	ReLU	128x4x1x8	128x1x293
2	Norm	-	-	128x1x293
3	Dropout	-	0.1	128x1x293
4	Conv	ReLU	128x128x1x8	128x1x286
5	Norm	-	-	128x1x286
6	Dropout	-	0.1	128x1x286
7	Flatten	-	-	36608
8	Dense	linear	256	256
9	Dropout	-	0.1	256
10	Dense	linear	128	128
11	Dense	Softmax	2	2
