# Supplementary material for: The impact of different negative training data on regulatory sequence predictions
Source: PLoS One. 2020 Dec 1;15(12):e0237412. doi: 10.1371/journal.pone.0237412 (PMC7707526; doi:10.1371/journal.pone.0237412)
Supplement: S5 Table — Ten CNN models of the 2conv2norm architecture were trained each on DHS datasets (positive) and corresponding negative sets of k-mer shuffled sequences (k = 2, k = 7) or genomic background sequences (tGC = 0.02) for A549 or MCF-7 cells. A549 and MCF-7 cell lines are represented in our data with two training datasets each, which are labeled as A and B, respectively. Model performance was evaluated based on recall for hold-out sets (chromosome 8). The table summarizes mean and standard deviation across ten trained models. There are seven different hold-out sets derived from different cell lines and we assess model generalization across cell-types. Datasets are named according to S1 Table. Respective results for the gkm-SVM models are available Table 1, results for CNN models of 4conv2pool4norm architecture are available in S6 Table. (PDF) [file pone.0237412.s022.pdf]

S5 Table: 2conv2norm recall for regulatory sequence prediction for different cell lines. Ten CNN models of the 2conv2norm architecture were trained each on DHS datasets (positive) and corresponding negative sets of k-mer shuffled sequences (k=2, k=7) or genomic background sequences (t_{GC}=0.02) for A549 or MCF-7 cells. A549 and MCF-7 cell lines are represented in our data with two training datasets each, which are labeled as A and B, respectively. Model performance was evaluated based on recall for hold-out sets (chromosome 8). The table summarizes mean and standard deviation across ten trained models. There are seven different hold-out sets derived from different cell lines and we assess model generalization across cell-types. Datasets are named according to S1 Table. Respective results for the gkm-SVM models are available Table 1, results for CNN models of 4conv2pool4norm architecture are available in S6 Table.

		Model											
		Shuffled (*k*=2)				Shuffled (*k*=7)				Genomic background (t_{GC}=0.02)			
		A549 (A)	A549 (B)	MCF-7 (A)	MCF-7 (B)	A549 (A)	A549 (B)	MCF-7 (A)	MCF-7 (B)	A549 (A)	A549 (B)	MCF-7 (A)	MCF-7 (B)
Recall (test set)	A549 (A)	0.890 ±0.036	0.840 ±0.09	0.871 ±0.035	0.872 ±0.061	0.692 ±0.021	0.633 ±0.026	0.678 ±0.017	0.655 ±0.012	0.770 ±0.050	0.609 ±0.082	0.740 ±0.041	0.677 ±0.084
	A549 (A)	0.871 ±0.038	0.846 ±0.086	0.856 ±0.036	0.859 ±0.062	0.610 ±0.017	0.600 ±0.024	0.596 ±0.015	0.578 ±0.01	0.753 ±0.046	0.665 ±0.079	0.732 ±0.038	0.671 ±0.075
	HeLa-S3	0.865 ±0.042	0.825 ±0.094	0.853 ±0.041	0.854 ±0.071	0.634 ±0.024	0.598 ±0.024	0.636 ±0.016	0.604 ±0.013	0.698 ±0.058	0.566 ±0.084	0.688 ±0.045	0.619 ±0.097
	HepG2	0.815 ±0.050	0.796 ±0.103	0.808 ±0.046	0.822 ±0.073	0.511 ±0.024	0.519 ±0.024	0.511 ±0.022	0.503 ±0.011	0.601 ±0.059	0.490 ±0.077	0.605 ±0.05	0.552 ±0.084
	K562	0.855 ±0.045	0.813 ±0.099	0.831 ±0.044	0.839 ±0.072	0.673 ±0.021	0.636 ±0.027	0.648 ±0.019	0.633 ±0.011	0.650 ±0.057	0.503 ±0.080	0.608 ±0.048	0.566 ±0.085
	MCF-7 (A)	0.864 ±0.042	0.819 ±0.097	0.886 ±0.035	0.883 ±0.061	0.642 ±0.023	0.598 ±0.026	0.684 ±0.016	0.655 ±0.013	0.713 ±0.057	0.555 ±0.087	0.797 ±0.039	0.724 ±0.085
	MCF-7 (B)	0.867 ±0.041	0.822 ±0.097	0.891 ±0.033	0.891 ±0.058	0.653 ±0.023	0.610 ±0.025	0.696 ±0.016	0.674 ±0.01	0.742 ±0.052	0.590 ±0.085	0.822 ±0.039	0.764 ±0.078
