# Supplementary material for: The impact of different negative training data on regulatory sequence predictions
Source: PLoS One. 2020 Dec 1;15(12):e0237412. doi: 10.1371/journal.pone.0237412 (PMC7707526; doi:10.1371/journal.pone.0237412)
Supplement: S6 Table — Ten CNN models of the 4conv2pool4norm architecture were trained each on DHS datasets (positive) and corresponding negative sets of k-mer shuffled sequences (k = 2, k = 7) or genomic background sequences (tGC = 0.02) for A549 or MCF-7 cells. A549 and MCF-7 cell lines are represented in our data with two training datasets each, which are labeled as A and B, respectively. Model performance was evaluated based on recall for hold-out sets (chromosome 8). The table summarizes mean and standard deviation across ten trained models. There are seven different hold-out sets derived from different cell lines and we assess model generalization across cell-types. Datasets are named according to S1 Table. Respective results for the gkm-SVM models are available Table 1, results for CNN models of 2conv2norm architecture are available in S5 Table. (PDF) [file pone.0237412.s023.pdf]

S6 Table: 4conv2pool4norm recall for regulatory sequence prediction for different cell lines. Ten CNN models of the 4conv2pool4norm architecture were trained each on DHS datasets (positive) and corresponding negative sets of k-mer shuffled sequences (k=2, k=7) or genomic background sequences (t_{GC}=0.02) for A549 or MCF-7 cells. A549 and MCF-7 cell lines are represented in our data with two training datasets each, which are labeled as A and B, respectively. Model performance was evaluated based on recall for hold-out sets (chromosome 8). The table summarizes mean and standard deviation across ten trained models. There are seven different hold-out sets derived from different cell lines and we assess model generalization across cell-types. Datasets are named according to S1 Table. Respective results for the gkm-SVM models are available Table 1, results for CNN models of 2conv2norm architecture are available in S5 Table.

		Model											
		Shuffled (*k*=2)				Shuffled (*k*=7)				Genomic background (t_{GC}=0.02)			
		A549 (A)	A549 (B)	MCF-7 (A)	MCF-7 (B)	A549 (A)	A549 (B)	MCF-7 (A)	MCF-7 (B)	A549 (A)	A549 (B)	MCF-7 (A)	MCF-7 (B)
Recall (test set)	A549 (A)	0.887 ±0.034	0.868 ±0.032	0.869 ±0.038	0.860 ±0.030	0.667 ±0.017	0.614 ±0.022	0.639 ±0.027	0.621 ±0.025	0.840 ±0.034	0.744 ±0.035	0.795 ±0.033	0.742 ±0.055
	A549 (A)	0.867 ±0.033	0.880 ±0.031	0.856 ±0.037	0.847 ±0.032	0.581 ±0.017	0.575 ±0.016	0.557 ±0.024	0.545 ±0.022	0.813 ±0.031	0.795 ±0.029	0.784 ±0.028	0.735 ±0.051
	HeLa-S3	0.858 ±0.04	0.856 ±0.038	0.849 ±0.047	0.835 ±0.025	0.611 ±0.017	0.575 ±0.021	0.598 ±0.031	0.576 ±0.024	0.773 ±0.041	0.694 ±0.033	0.756 ±0.040	0.691 ±0.060
	HepG2	0.806 ±0.046	0.818 ±0.04	0.805 ±0.044	0.800 ±0.041	0.476 ±0.024	0.493 ±0.019	0.468 ±0.030	0.465 ±0.024	0.685 ±0.040	0.626 ±0.040	0.668 ±0.038	0.617 ±0.063
	K562	0.848 ±0.04	0.833 ±0.038	0.825 ±0.044	0.819 ±0.041	0.648 ±0.021	0.609 ±0.025	0.609 ±0.026	0.595 ±0.026	0.729 ±0.041	0.635 ±0.044	0.674 ±0.037	0.627 ±0.065
	MCF-7 (A)	0.857 ±0.04	0.845 ±0.038	0.888 ±0.038	0.875 ±0.026	0.621 ±0.017	0.577 ±0.022	0.651 ±0.025	0.631 ±0.024	0.790 ±0.038	0.700 ±0.036	0.858 ±0.029	0.801 ±0.049
	MCF-7 (B)	0.864 ±0.037	0.853 ±0.035	0.894 ±0.036	0.886 ±0.026	0.628 ±0.018	0.590 ±0.022	0.666 ±0.027	0.651 ±0.025	0.816 ±0.037	0.733 ±0.035	0.876 ±0.028	0.839 ±0.043
