# Supplementary material for: The impact of different negative training data on regulatory sequence predictions
Source: PLoS One. 2020 Dec 1;15(12):e0237412. doi: 10.1371/journal.pone.0237412 (PMC7707526; doi:10.1371/journal.pone.0237412)
Supplement: S7 Table — Models were trained on DHS sequences (positive) with corresponding sets of negative sequences and tested on a set of tissue-specific chromosome 21 test set. For each classifier two different negative training sets are compared; sequences were either chosen from genomic background (tGC = 0.1) or generated by shuffling positive sequences and preserving k-mer counts (k = 7). AUROC value was calculated to compare model performance. (PDF) [file pone.0237412.s024.pdf]

S7 Table: AUROC values for tissue-specific regulatory sequence prediction on validation sets. Models were trained on DHS sequences (positive) with corresponding sets of negative sequences and tested on a set of tissue-specific chromosome 21 test set. For each classifier two different negative training sets are compared; sequences were either chosen from genomic background (t_{GC}=0.1) or generated by shuffling positive sequences and preserving k-mer counts (k=7). AUROC value was calculated to compare model performance.

training cell line	2conv2norm		4conv2pool4norm		gkm-SVM	
	genomic backgr. (t_{GC}=0.1)	shuffled (k=7)	genomic backgr. (t_{GC}=0.1)	shuffled (k=7)	genomic backgr. (t_{GC}=0.1)	shuffled (k=7)
A549	0.532	0.545	0.516	0.517	0.476	0.540
HeLa-S3	0.675	0.591	0.669	0.584	0.691	0.646
HepG2	0.591	0.503	0.574	0.517	0.589	0.548
K562	0.647	0.633	0.633	0.627	0.629	0.641
MCF-7	0.620	0.540	0.640	0.558	0.693	0.668
