# Supplementary material for: The impact of different negative training data on regulatory sequence predictions
Source: PLoS One. 2020 Dec 1;15(12):e0237412. doi: 10.1371/journal.pone.0237412 (PMC7707526; doi:10.1371/journal.pone.0237412)
Supplement: S8 Table — Models were trained on DHS sequences (positive) with corresponding sets of negative sequences and tested on a set of tissue-specific chromosome 21 test set. For each classifier two different negative training sets are compared; sequences were either chosen from genomic background (tGC = 0.1) or generated by shuffling positive sequences and preserving k-mer counts (k = 7). AUPRC value was calculated to compare model performance. (PDF) [file pone.0237412.s025.pdf]

S8 Table: AUPRC values for tissue-specific regulatory sequence prediction on validation sets. Models were trained on DHS sequences (positive) with corresponding sets of negative sequences and tested on a set of tissue-specific chromosome 21 test set. For each classifier two different negative training sets are compared; sequences were either chosen from genomic background (t_{GC}=0.1) or generated by shuffling positive sequences and preserving k-mer counts (k=7). AUPRC value was calculated to compare model performance.

training cell line	random performance	2conv2norm		4conv2pool4norm		gkm-SVM	
		genomic backgr. (t_{GC}=0.1)	shuffled (k=7)	genomic backgr. (t_{GC}=0.1)	shuffled (k=7)	genomic backgr. (t_{GC}=0.1)	shuffled (k=7)
A549	0.006	0.006	0.006	0.007	0.006	0.006	0.006
HeLa-S3	0.345	0.503	0.427	0.475	0.429	0.537	0.491
HepG2	0.072	0.114	0.073	0.096	0.072	0.104	0.094
K562	0.254	0.364	0.343	0.342	0.336	0.366	0.370
MCF-7	0.087	0.124	0.104	0.130	0.105	0.170	0.165
