# Supplementary material for: The impact of different negative training data on regulatory sequence predictions
Source: PLoS One. 2020 Dec 1;15(12):e0237412. doi: 10.1371/journal.pone.0237412 (PMC7707526; doi:10.1371/journal.pone.0237412)
Supplement: Attachment — Submitted filename: reviewer_responses.pdf [file pone.0237412.s026.pdf]

Editors' comments:

[...]

After careful consideration, we feel that it has merit but does not fully meet PLOS ONE’s publication criteria as it currently stands. Therefore, we invite you to submit a revised version of the manuscript that addresses the points raised during the review process.

As you can appreciate from the attached reports, both reviewers agreed that this is an important study, and that is was generally well conducted, but they have made suggestions for improving certain aspects. In particular, we feel that it would be important to address the major points raised by reviewer #2 regarding the mixing of promoters and enhancers in the analyses, and performing cross-cell line tests using a genomic background negative set.

Please submit your revised manuscript by Oct 02 2020 11:59PM. If you will need more time than this to complete your revisions, please reply to this message or contact the journal office at plosone@plos.org. When you're ready to submit your revision, log on to https://www.editorialmanager.com/pone/ and select the 'Submissions Needing Revision' folder to locate your manuscript file.

Please include the following items when submitting your revised manuscript:

- A rebuttal letter that responds to each point raised by the academic editor and reviewer(s). You should upload this letter as a separate file labeled 'Response to Reviewers'.
- A marked-up copy of your manuscript that highlights changes made to the original version. You should upload this as a separate file labeled 'Revised Manuscript with Track Changes'.
- An unmarked version of your revised paper without tracked changes. You should upload this as a separate file labeled 'Manuscript'.

[...]
Miguel Branco
[...]

1. Please ensure that your manuscript meets PLOS ONE's style requirements, including those for file naming. The PLOS ONE style templates can be found at https://journals.plos.org/plosone/s/file?id=wjVg/PLOSOne_formatting_sample_main_body.pdf and https://journals.plos.org/plosone/s/file?id=ba62/PLOSOne_formatting_sample_title_authors_affiliations.pdf

Done

2.Thank you for stating the following in the Acknowledgments Section of your manuscript:
[This work was supported by the Berlin Institute of Health and Charité – Universitätsmedizin Berlin. The funder had no involvement in study design; in the collection, analysis and interpretation of data; in the writing of the report; and in the decision to submit the article for publication.]
We note that you have provided funding information that is not currently declared in your Funding Statement. However, funding information should not appear in the Acknowledgments section or other areas of your manuscript. We will only publish funding information present in the Funding Statement section of the online submission form.

Please remove any funding-related text from the manuscript and let us know how you would like to update your Funding Statement. Currently, your Funding Statement reads as follows:
[The author(s) received no specific funding for this work.]

We removed the mentioned sentences from the acknowledgments.

Reviewers' comments:

[...]

Reviewer #1: Krutzfeldt et al. investigate 3 machine learning models (2 CNN and one gkm-SVM) on predicting accessible regions and enhancers in human cell lines from DNA sequence alone. In particular, the authors focused on selection of different negative sequences and how that affects the model performs. The authors are able to identify with high accuracy accessible regions of the genome. Nevertheless, their results are relatively poor for cell specific accessible regions and enhancers, which is expected for DNA sequence only models. Furthermore, the authors show that using one model to select the negative set (genomic background) provides better and robust results in most test scenarios. The paper is well written and presents important results.

We thank the reviewer for the positive review of our work.

There are some points that authors would need to address before I could recommend this paper for publication:

1. An explanation of CNNs and gkm-SVMs could help readers that are unfamiliar with these machine learning algorithms.

We thank the reviewer for this comment. We have added the following descriptions to the manuscript, even though we would not expect someone would read our manuscript without any prior exposure to these two types of models.

Support vector machines are a class of machine learning algorithms mainly used for classification problems, where they find a projection in a high-dimensional space with an optimal decision boundary (hyperplane). In the case of gkm-SVMs, SVM classifiers are applied to strings representing gapped k-mers, i.e. oligonucleotides of fixed size k from a library of DNA sequences that includes non-informative positions named gaps. A convolutional neural network is a class of deep neural networks that takes advantage of hierarchical patterns in data and assembles complex patterns (using multiple convolution and pooling layers) from simpler, smaller patterns, so-called convolutional kernels.

2. Line 66-74, the authors mention that there is a strong link between TF binding and DNA accessibility. While this would be the case for many TFs, it is not generally true (see https://www.sciencedirect.com/science/article/abs/pii/S1097276520303579?via%3Dihub, https://science.sciencemag.org/content/368/6498/1460 or https://www.biorxiv.org/content/10.1101/666446v1.abstract). I think the authors should discuss about these different types of mechanisms.

While these examples exist (and are frequently summarized as the effects of pioneer factors), it does not seem a major effect or at least from our experience is currently not widely appreciated

as such [e.g. from ENCODE https://doi.org/10.1038/nature28171]. Throughout the manuscript, we are citing major papers that support the view of a majority of TFs binding to chromatin-free DNA. We added two of the references suggested by the reviewer and rephrased the first sentence of the section as follows:

With some notable exceptions [6,7], there is a strong link between transcription factors (TFs) binding to regulatory elements and general DNA accessibility, i.e. open chromatin.

References:

6. Michael AK, Grand RS, Isbel L, Cavadini S, Kozicka Z, Kempf G, et al. Mechanisms of OCT4-SOX2 motif readout on nucleosomes. Science. 2020;368: 1460–1465. doi:10.1126/science.abb0074

7. Lerner J, Gomez-Garcia PA, McCarthy RL, Liu Z, Lakadamyali M, Zaret KS. Two-Parameter Mobility Assessments Discriminate Diverse Regulatory Factor Behaviors in Chromatin. Mol Cell. 2020;79: 677-688.e6. doi:10.1016/j.molcel.2020.05.036

3. Line 75-85, the DNA sequence can predict TF binding sites, but many TFs bind in a concentration dependent manner (e.g. https://journals.plos.org/plosgenetics/article?id=10.1371/journal.pgen.1001290, https://journals.plos.org/plosgenetics/article?id=10.1371/journal.pgen.1003571 or https://academic.oup.com/nar/article/43/1/84/2903035).

We thank the reviewer for pointing us to these manuscripts and suggesting to us the inclusion of concentration dependent binding effects in our general introduction of transcription factor binding. This topic seems a little too complex for a general introduction though, as it is also dependent on the number of potential/accessible binding sites and not only the protein-level of the TF. Instead, we decided to highlight the cell-type specific expression of TFs earlier in the text:

TFs are known to have different binding affinities to DNA sequences, to bind preferentially to a specific set of short nucleotide sequences named binding motifs [13] and to vary in their cell-type expression [9].

References:

9. The ENCODE Project Consortium. An integrated encyclopedia of DNA elements in the human genome. Nature. 2012;489: 57–74.

13. Boeva V. Analysis of genomic sequence motifs for deciphering transcription factor binding and transcriptional regulation in eukaryotic cells. Frontiers in Genetics. 2016;7: 24.

4. "The tolerance for differences in repeat ratio and relative sequence length were set to 0, but the tolerance for differences in GC content was varied for different training datasets (tGC={0.02, 0.05, 0.1})." – The authors need to explain why this was varied.

GC content was underexplored in the original study and we suspected an effect of GC content on gene expression due to the known GC bimodality of promoters (CpG island associated vs others). Similar effects are therefore expected for regulatory regions in general. We added the following sentence to the method description:

“We explored a range of GC tolerances as GC content has been associated with gene expression regulation and the original study [17] did not explore this parameter.”

References:

17. Ghandi M, Mohammad-Noori M, Ghareghani N, Lee D, Garraway L, Beer MA. gkmSVM: an R package for gapped-kmer SVM. Bioinformatics. 2016;32: 2205–2207.

5. “The parameter k which indicates the size of the preserved k-mers was varied for different datasets (k=[1,7])” – Again, it the authors need to explain why this was varied.

This parameter was varied as different studies motivate different values of k based on different average length of TF motifs or the need of destroying such motifs in shuffled sequences. We did not see a consensus from previous literature and tested the reported range. We include the following sentence in the method descriptions now:

"Here, we explore a range of k as we were unable to identify a consensus value from prior literature [22,27–30]."

References:

22. Alipanahi B, Delong A, Weirauch MT, Frey BJ. Predicting the sequence specificities of DNA- and RNA-binding proteins by deep learning. Nature Biotechnology. 2015;33: 831–838.
27. Zeng H, Hashimoto T, Kang DD, Gifford DK. GERV: a statistical method for generative evaluation of regulatory variants for transcription factor binding. Bioinformatics. 2016;32: 490–496. doi:10.1093/bioinformatics/btv565
28. Zhou T, Shen N, Yang L, Abe N, Horton J, Mann RS, et al. Quantitative modeling of transcription factor binding specificities using DNA shape. Proc Natl Acad Sci USA. 2015;112: 4654–4659. doi:10.1073/pnas.1422023112
29. Shen Z, Bao W, Huang D-S. Recurrent Neural Network for Predicting Transcription Factor Binding Sites. Sci Rep. 2018;8: 15270. doi:10.1038/s41598-018-33321-1
30. Arvey A, Agius P, Noble WS, Leslie C. Sequence and chromatin determinants of cell-type-specific transcription factor binding. Genome Res. 2012;22: 1723–1734. doi:10.1101/gr.127712.111

6. For liver enhancer activity data, have the enhancers from Inoue et al. (2017) been cross validated by another method? For example with STARR-seq from https://genomebiology.biomedcentral.com/articles/10.1186/s13059-018-1473-6 .

Yes, there happens to be a bioRxiv manuscript (https://www.biorxiv.org/content/10.1101/576405v1) describing the read-out of these same elements (different library, with different barcode associations) in 3'/5' lentiMPRA, in plasmid-based MPRAs and in STARR-seq experiments. Correlations between different assays are very high for these kinds of assays (Fig 2, ORI vs 5'/3' WT, Pearson correlation 0.74).

7. “We evaluated models on their respective hold-out and additionally the models trained on shuffled data on hold-out using genomic background sequences as negative sets” – This makes sense after reading and re-reading it a few times but it isn’t immediately clear. I’d recommend finding a way to re-word it.

We thank the reviewer for pointing out that this sentence is difficult to understand. We rephrased the sentence to read as follows:

“We evaluated models on their respective hold-out sets. Additionally, models trained on shuffled data were evaluated on hold-outs using genomic background sequences as negative sets.”

8. It wasn’t particularly clear exactly what information the authors use as an input. In Min et al. (2016) they use a 300bp sequence as an input. As they are using DeepEnhancer and a smaller network based on the DeepEnhancer architecture I assumed this was the same, but then I wondered how you dealt with merging of positive bins. In the materials and methods section you state that positive DNA-seq datasets were created where: “Multiple technical replicates were merged into one file per experiment, combining overlapping (minimum of 1 bp) or adjacent sequences into a single spanning sequence”. When two regions are merged, is a new centre is calculated between the two, then the input region is classed as 300bp around the new centre?

Yes, this is correct. We extended the method description of the positive training data to make it explicit.

“DHS regions were defined 300 bp around the center of the narrow peaks[/merged segments] and reference genome sequences used (GRCh38 patch release 7, GRCh38.p7).”

9. Do you treat a peak within 150bp of the centre of another peak as a separate instance? This peak is not overlapping and is not adjacent, but it would contain some of the same sequence.

As follows from the earlier description, if the narrow peak calls do not overlap/are not adjacent, these are two separate peaks. Considering an average peak size of 150 bp for the ENCODE narrow peaks, yes, it is possible that flanking sequences of two selected sequences may overlap. This is to be expected and has no downstream consequences.

10. Citations for some Biocoductor packages are missing. Cite the recommended papers.

Thank you for spotting this. However, we found only one Bioconductor package (Biostrings) that is currently not cited. We included the missing citation for Biostrings and BSgenome.Hsapiens.UCSC.hg38.masked.

11. Line 233: “First models were tested on validation sets to identify best parameters for generating the negative training set based only on recall measures.”. Maybe I misunderstood something, but shouldn’t the training be done on the training set not the validation set?

We are sorry, but you must have misunderstood. The sentence refers to testing the models.

12. “However, models trained on highly shuffled data perform significantly better than models trained on genomic background data; potentially the result of an improper evaluation on varying compositions of the validation sets using different negative data” – Have the authors compared the compositions of the validation sets to test this?

Yes, we did. We were discussing the results in lines 338-348 of the originally submitted manuscript. The major finding from this section: “We observe that 8-mers in the genomic background negative sets are on average more frequent than 8-mers from DHS sites (positive sets) and those are more frequent than 8-mers from shuffled negative sequences (S10A Figure).”

13. When mentioning Figure S10, A and B are mentioned in the text but not C. C is the Z-score of the larger CNN (which looks much the same as B) but it’s just worth pointing out.

Thank you for spotting this. Now, we are also referring to Figure S10 C.

14. Is there generalisation data for the CNNs as well as the Gkm-SVM models? (as in Table 1)

We had not included that, but are including it now as tables S5 for 2conv2norm and S6 for 4conv2pool4norm. Existing tables were renamed to S7 and S8, respectively. We see very similar performance for models trained on k=2 shuffles, with best performance for the same cell-type and only slightly reduced performance on all other cell types. For comparison, average recall values across all comparisons for gkm-SVM, 2conv2norm, and 4conv2pool4norm are 0.86, 0.85 and 0.85, respectively.

In response to the other reviewer's request, we also included k=7 as well as the genomic background in all tables. The performance of k=7 is reduced for this task (average recall of 0.49, 0.62, and 0.59 for gkm-SVM, 2conv2norm and 4conv2pool4norm, respectively), as also previously described for task 1. Models trained on genomic background perform similarly for gkm-SVM (average recall of 0.75) and 4conv2pool4norm (average recall of 0.74), but show lower performance for the smaller CNN architecture (2conv2norm, average recall of 0.66).

15. For S5 and S6 probably shouldn't be mentioned until after it is explained why k=7 is used. After the previous section explaining that k=2 was close to optimal it seems strange to jump to the new figure without the explanation that comes further down the paragraph.

We agree with the reviewer and reordered sentences in the paragraph to make the results and our decisions based on them clear:

"Again, we first tested parameter choice on a validation set (chromosome 21 hold-out). Since HeLa-S3 models performed best, we focus the presentation of results on this cell line For the genomic background set, we chose again the most stringent parameter (t_{GC}=0.02) as models trained using genomic background showed similar performance independent of the GC content tolerance (S12 Figure). For shuffled sequences, we picked k=7 based on precision recall (S13 Figure). Model performance tends to increase with higher size of preserved k-mers in shuffled sequences. The high value of k preserves a number of TFBS motifs (46±2 motifs per 300 bp) similar to the positive set (47±2 motifs per 300 bp, S7 Figure), suggesting that presence of tissue-specific factors as well as relative positioning may be most critical for model performance. We notice that performance is considerably reduced compared to the first task and see big differences regarding model performance across different training cell lines (S7 and S8 Tables)."

16. It isn't particularly clear how models were trained on enhancers. Are there annotated datasets for those cell lines?

We did not change our DHS training datasets when <u>testing</u> on liver enhancers. All analyses are based on the same training data and experimental design, our study focuses on relative differences in model performance between the parameters tested. We discuss the mixture of elements (promoter/enhancer) for training in our response to reviewer 2.

17. As a general comment, it is easier to read Figure S1 rather than S1 Figure.

This is the PLOS One citation style for figures. We cannot implement this change.

18. Line 305: I would reference https://doi.org/10.1016/j.tig.2009.08.003 about TF motif size in eukaryotic genomes, which could explain why 7-Kmer would overlap with many TF motifs.

While we appreciate the reviewer's suggestion, we consider this an unusual reference for the length of TF motifs. We decided to not cite it for the result section of our manuscript.

19. K-mer 1 is the best, but authors use K-mer 2. I think the authors should include K-mer 1 in their manuscript in order to allow the reader to judge the performance of the model.

Results for other kmer sizes are already available in S5 and S6 Figure and referenced in the respective paragraph. Further, we explain in the text that we show k=2 due to its wider usage. We therefore did not implement any changes in response to the reviewer's comment.

20. Figure 1 needs better explanation. It took me a while to understand the difference between top and bottom panels and red/blue colours.

We adjusted the figure and legend in concordance with the suggestions from Reviewer 2 (see below).

21. Lines 338-348: needs to be explained better. It was difficult to follow.

We slightly edited the paragraph, we hope the results are now easier to follow.

"[…] we analyzed 8-mer frequency in the different test data set classes (i.e. DHS sites, genomic background, and shuffled sequences) as well as 8-mers prioritized in the first convolutional layer of our CNN models. We compared these 8-mers based on their genomic frequency across the human genome. We observe that 8-mers in the genomic background negative sets are on average more frequent than 8-mers from DHS sites (positive sets) and those are more frequent than 8-mers from shuffled negative sequences (S10A Figure). [...]"

22. In Figure S10A, k-mer shuffle plots several blue lines; they look similar with DHS and genomic background except for one outlier. Can authors clarify this?

Blue lines represent the different k-mer sizes of the shuffles (as described in the legend). At the left and right tails of the plotted distributions, red/blue lines are (independent of k) on opposite sides of the DHS distribution. Thus, they could be separated using machine learners, despite an incomplete separation in the middle part of the plot.

23. Table 1: The authors need to show a Venn diagram with the overlap of DHS between the different cell lines, so the reader can judge these results. The reason the model generalises so well might be because the DHS in the different cells overlap significantly.

The reviewer suggests that a large overlap of active DHS sites might underlie the generalization performance. The suggested Venn diagrams for 7 sets seem not useful. Instead, we present the pairwise overlap between training, validation and test sets for the different cell types in a Figure S11 (renaming all subsequent supplemental figures). We note that overlaps are generally low (≤ 0.34), even for data sets of the same cell-type (≤ 0.53). We highlight this result in the text:

24. Lines 397-404: The authors need to discuss that the reason the model doesn't perform so well on cell specific data is because while DNA sequence is the same, there something else that controls tissue

specific regions. Their method is better at predicting constitutive DHS and enhancers and will suffer when predicting tissue specific enhancer.

Our manuscript looks at DHS sites in general. We do not distinguish between enhancers or promoters. As can be seen from Tables S1 and S2, for HeLa-S3 and K562 almost half of all DHS sites are cell-type specific, while for other cell lines this proportion is as low as approximately 10% (A549). As can be seen from Table 1, a model trained on 10% specific sequences (A549) does not perform that much different from a model where about 30% are specific (MCF-7) and no relevant differences are observed in generalization performance across cell-lines. As pointed out above, we are now also reporting pairwise peak overlaps for task 1 (S11 Figure).

25. Authors should include a graph with the run time of the different models to allow quantitative assessment of the model.

We did not record runtimes. It is widely appreciated that gkm-SVM models take long to train and we included two citations in the manuscript:

This is inline with gkm-SVMs being the simpler machine learning approach (despite being slower in their current implementation [33,48]) and we see this as a cautionary reminder to keep models simple, especially if training data is limited.

References:

33. Lee D. LS-GKM: a new gkm-SVM for large-scale datasets. Bioinformatics. 2016;32: 2196–2198.
48. Chen L, Fish AE, Capra JA. Prediction of gene regulatory enhancers across species reveals evolutionarily conserved sequence properties. PLOS Computational Biology. 2018;14: e1006484. doi:10.1371/journal.pcbi.1006484

Further, we now include the information that we did not run the larger gkm-SVM models that would have taken more than 3 weeks to train (text in reference to Fig 4):

"Results are presented in Fig 4. Model performance of gkm-SVM classifier seems very stable across training set sizes and repeated training runs, but due to runtime (more than 3 weeks) we did not test more than 350,000 positive training examples."

26. It is not clear why for different cell types different optimal K-mers are used. Can the authors explain this?

We identified different optimal k values for different tasks, not for different cell-types.

27. Lines 431-437: I assumed that the data is available only for HepG2, but what data is used for K562, A549 and HeLa?

The reviewer is correct, the experimental read-outs are only available for HepG2. We are using models trained on DHS sites from different cell-types though (Table S1). As we discuss in lines 435-437, we observe that even a mixed data (i.e. pool of DHS sites from several cell-types) set shows an improved performance; highlighting the training data limitation of our HepG2 model.

28. The performance to predict enhancers is relatively poor, but within the results of other papers. The authors should make this clear.

Our manuscript looks at DHS sites in general (as others have done before) and it was not our goal to present an improved sequence based predictor for enhancers or other regulatory regions. Our work predominantly discusses the effects of choosing appropriate (negative) training data sets. We have not performed an extensive hyperparameter search, nor did we explore multi-task or multi-modal models which are expected to show an increased performance for the mentioned prediction tasks. We believe, we were very careful in using the right terminology throughout the manuscript. However, to clarify this again, we included the following sentence in the first paragraph of the conclusions:

We use open chromatin regions as a general proxy for regulatory sequences and do not differentiate between promoters or enhancers/silencers.

Reviewer #2: In the manuscript by Krutzfeldt et al., the authors assess the impact of different negative training sets on regulatory sequence prediction. Three models (one gkm-SVM and two CNN) are trained using DNase hypersensitive sites as positive set and either genomic background or shuffled sequences as negative set. Effects of the choice of background are compared across three tasks: hold-out prediction regulatory sequences, cell type specific hold-out prediction and prediction of enhancer activity (correlation with experimental data). The authors find that shuffled sequences contain rare/artificial 8-mers/motifs which are used by the learners which makes models perform worse on biologically more relevant tasks.

This is a wonderful example of how the definition of 'background' (i. e. the baseline you compare whatever you are interested in against) has a large influence on the results – which is a common theme in bioinformatics. Unfortunately, the question which background to choose is often not given enough thought. Therefore, studies like this are very relevant and can be eye opening. With machine learning methods becoming more accessible and more widely used information on what to watch out for is pertinent. The manuscript contains relevant data, the rationale is well explained and methods are sufficiently described. It is written in an easy to follow style.

We thank the reviewer for this thoughtful and kind review of our work.

Major points for consideration

It seems likely that looking at all DHS sites together results in a mix of functional biological elements, presumably dominated by enhancers and promoters. These are likely defined by different sequence features and we could envision a scenario in which promoter activity might be strongly influenced by presence/absence of a TFBS (strong sequence feature), while enhancer activity might be more dependent on histone modifications (weak sequence feature).

We appreciate the reviewers' comment and totally agree that these differences may or are even likely to exist. We want to highlight though, that our manuscript focuses on a relative comparison and that we are not attempting to create a superior model for the prediction of open chromatin regions, enhancers or promoters. We use the prediction of open chromatin as a mere example of a relevant prediction task. As mentioned also to the other reviewer, we have not performed an extensive hyperparameter search, nor did we explore multi-task or multi-modal models which are expected to show an increased performance for the mentioned prediction tasks.

Thus, it seems possible that the method of negative region definition (shuffling or genomic background) may impact differently on different functional elements. If this were the case, it would affect interpretation of the results at several points throughout the manuscript. The authors mention one example in the context of GC matching (p18, lines 503-516, Figure S16): The distribution of GC content of the DHS sites is clearly bimodal. It seems likely that the less CG rich peak mainly contains enhancers and non-CGI promoters while the GC rich peak is dominated by CGI promoters. Sequence features learned by a model would probably be very different if learned on those three subgroups and could be affected quite differently by choice of negative set. I am wondering if a very crude distinction of DHS sites into promoter distal (putative enhancers) and CGI and non-CGI promoter proximal would result in substantially improved models and how these would be affected by different negative data.

We agree that the bimodality is likely due to CpG island promoters, as others have described unimodal distributions for enhancers (e.g. https://doi.org/10.1534/genetics.118.301116). This previous work describes a very long tailed GC distribution though, which would also not be correctly matched by the used package sampling the genomic background. Thus, the general need of matching an empirical GC distribution rather than mean and standard deviation holds. We refer to that in our conclusions:

"With the genomic background method applied here [17], we notice that GC matching should be improved to closely reproduce the continuous GC density distribution of the positive set rather than a mean and standard deviation."

Reference:

17. Ghandi M, Mohammad-Noori M, Ghareghani N, Lee D, Garraway L, Beer MA. gkmSVM: an R package for gapped-kmer SVM. Bioinformatics. 2016;32: 2205–2207.

Even though not explicitly part of our study, we do not expect that genomic sequences would work better for one type of regulatory sequence but not the other. We identified that learners included rare motifs from artificial sequence sets as the main disadvantage of shuffled sequences. This observation will hold for any subsample of open chromatin regions. Further, splitting up DHS peaks into different types of regulatory elements will reduce training data set size, which we identified as a major bottleneck in training (Fig. 4). In our analysis, model performance improved even when adding DHS sites from another cell-type and despite the low overlap of DHS sites between cell-types.

P 15 line 358, Table 1: In previous paragraphs, the authors have convincingly shown that shuffled 2-mers contain rare/artificial motifs and that the learners are affected by these. Here, these models based on shuffled regions are used to compare performance across cell types. However, since part of the learning is based on the distinction between biological and artificial, performance of a model trained on one cell type is expected to be high in another (we assume that sequence composition doesn't change between cell lines). How does model performance generalise across cell lines when genomic background is used as negative sets? Does this still support the idea that organismal rather than tissue specific regulatory features are predicted? The point that models are strongly influenced by differences between biological and artificial sequences is really driven home by the data for tissue-specific regulatory region prediction: models trained on highly shuffled sequences are only marginally better than random (or not at all if k=1!). The comparison above therefore also needs to include data for higher k-mers (7-mers to be able to compare with the figures on tissue specific region prediction). I consider the comparison across cell-types for models trained on genomic background and 7-mer shuffles essential.

We have extended Table 1 to include results from using genomic background data, we also included another k (k=7) for the shuffled background data. As outlined before, best performance (average recall 0.86, k=2) in task 1 is observed for models trained on highly shuffled sequences. The performance of models trained using genomic background is somewhat reduced (average recall 0.75), while the performance of models of lightly shuffled sequences is considerably worse (average recall 0.49, k=7). As can be seen from S11 Figure, data sets show very little overlap of DHS sites across cell-types (overlap ≤ 0.34), even across experiments of the same cell-type (overlap up to 0.53). This might partially be due to incomplete sampling of all DHS sites in these experiments, however still highlights the ability of the models to generalize in identifying potential open chromatin regions.

We modified the section referencing Table 1 as follows:

"For A549 and MCF-7 cell lines with two available DHS sets from ENCODE, two separate models were trained and their performance on the test sets compared among all cell lines (Table 1). We see that performance generalizes well across diverse cell lines (e.g. breast, cervix, lung, liver cancer and leukemia), suggesting that organismal rather than tissue-specific active regulatory regions are predicted. As an example, Table 1 shows recall values for the gkm-SVM models trained on shuffled sequences (k=2) ranging from 0.79 to 0.88 for other cell types. This highlights the ability of models to generalize in identifying potential open chromatin regions, despite DHS peaks in training, validation and test showing very little overlap across cell-types (pairwise overlap of ≤ 0.34) or even across experiments of the same cell-type (pairwise overlap of up to 0.53, S11 Figure). As to be expected, models trained on A549 training sets perform best on A549 test sets (recall of 0.86 and 0.88, respectively) and MCF-7 models perform best on MCF-7 datasets (recall of 0.90 and 0.91, respectively)."

Further, we modified the legend of Table 1:

"Table 1: Recall of test set regulatory sequence prediction for different cell lines. Gkm-SVM models were trained on DHS datasets (positive) and corresponding negative sets of k-mer shuffled sequences (k=2, k=7) or genomic background sequences (t_{GC}=0.02) for A549 or MCF-7 cells; cell lines with two training datasets (A/B) each. Model performance was evaluated based on recall for hold-out sets (chromosome 8). There are seven different hold-out sets derived from different cell lines and we assess model generalization across cell-types. Best performance is observed for models trained on highly shuffled sequences (k=2), model performance is reduced when trained on genomic background, while the performance of models trained on lightly shuffled sequences (k=7) is considerably worse. Datasets are named according to S1 Table. Results for the CNN models (2conv2norm and 4conv2pool4norm) are available in S5 and S6 Table, respectively."

P16 line 306: Quantitative enhancer activity prediction: As mentioned above the DHS sites are a mix of functional elements, presumably mainly enhancer and promoter sequences. Therefore, the model will learn both enhancer and promoter sequence features, however, the experimental readout is only enhancer activity. This is bound to impact negatively on correlation, so maybe it's not so surprising that the correlation is so low. The differences in overall correlation when using different parameters (flexibility in GC content and k-mers) are very small and often the Spearman's rank correlation hovers around 0.1. This is such low correlation and such small changes, that I would be very reluctant to assign meaning to parameters different to before performing better in this task. This should be made clearer in the text. Moreover, it would be much more informative to plot the actual value pairs rather than just the value for the overall correlation. It might even reveal subgroups of value pairs, some with

better correlation than others. An idea might be to colour promoter proximal/distal or CGI/non-CGI data points differently to see if patterns emerge.

We assume that the reviewer refers to p18, lines 406ff. We agree with the reviewer that there is a general agreement that Pearson correlations below 0.5 (R^2 of 0.25) are not considered predictive models. This is based on a "variance explained by a linear model" argument. However, this does not mean that significant (Spearman) correlations below this value can not be interpreted, or that differences in correlation values have no meaning. Due to the simplicity of how correlations are calculated, they tend to be rather stable in these contexts and the relative performance of models can be evaluated using rank correlations (Spearman rather than Pearson). You can see the low variance observed in calculating Spearman correlation between 10 model training instances in Fig 3. We also like to point out that the publication we are comparing to reports the Spearman correlation of 0.276 (R^2 of 0.076). As it is possible to calculate confidence intervals for these values, we have added these confidence levels to Figures S14 and S15 (renamed from S13 and S14 due to the insertion of a supplementary figure). You can see that confidence intervals are different from 0 for most of the model parameters tested and that obtained correlation coefficients, despite being small, are significantly different between certain parameter choices.

Why were chromosome 21 and 8 chosen as validation and test sets, and why was this preferred over randomly selected positive and negative regions? Genomes are known to show chromosomal bias for genomic features and potential differences between training and test/validation chromosomes should be assessed, e. g. sequence composition, density of positive and negative regions, GC content, density of repetitive elements etc.

Chromosomes 8 and 21 are not known for compositional artifacts in terms of gene density, GC content, or repeat elements (in contrast to for example 17 and 19). The high similarity in overlaps within and between cell-types for train, validation and test data sets is also indicative for that (new Figure S11). By using chromosome hold-outs, we can reduce issues due to segmental duplications and highly similar sequences or due to neighboring peaks with overlapping training sequences. Similarly, the effects of correlated DHS sequences due interacting promoters/enhancers with shared binding motifs are reduced. Evaluation on hold-outs also prevents models to learn features of element density and regionality and simplifies comparisons of models between cell-types, experiments and model parameters. The smaller chromosome (21) is used for fast validation/parameter optimization, while the larger chromosome is used for testing.

Chromosome hold-outs were previously applied in several prior publications:
Expecto (https://doi.org/10.1038/s41588-018-0160-6),
DeepSEA (https://doi.org/10.1038/nmeth.3547),
NDEA (https://doi.org/10.1038/s41588-019-0420-0),
MtBNN (https://doi.org/10.1093/bioinformatics/btz767),
TBiNet (https://doi.org/10.1038/s41598-020-70218-4), or
DeepGMAP (https://doi.org/10.1371/journal.pone.0235748).

Minor points

P 13, line 257, Frequency distribution of 8-mers. While all other genomic sequences are based on GRCh38, this frequency analysis uses GRCh37 – presumably because it was reused from a previous study. Since these are both mature genome builds, it is probably fair to assume that the distribution

doesn't change significantly, but do the authors have any information on this? Also, the models include the sex chromosomes, while the frequency analysis doesn't. Does this matter?

We noticed that we are only presenting results from the test data set (chromosome 8), we removed the reference in the methods section regarding the training data. Since this is a relative analysis between the different "classes" in our input data, we did not expect to see a difference depending on how kmer frequencies are derived. However, we now repeated the analysis, using GRCh38 autosomes and gonosomes. We updated figures and descriptions for this analysis. The results are indistinguishable.

P 14 line 314 "potentially the result of an improper evaluation on varying compositions of the validation sets using different negative data." I don't understand the meaning of this sentence.

We are sorry for this confusing statement. This was meant as a cautionary note, that we will look at this result again in the next paragraph, where we discuss whether model performances can be compared across different negative data sets used for evaluation (and not just for training). We removed this half-sentence.

While explanations are in the text, I found Figure 1 quite confusing and clearer labels would help. The legend title should be something like "model trained on" as pink indicates that the model was trained on genomic background and blue indicates that the model was trained on shuffled sequences. Likewise, the labels for top and bottom panels should include more information, e. g. "negative regions in the test set: genomic background".

We appreciate your suggestions on Figure 1 and changed the figure accordingly. We hope it is much clearer now.

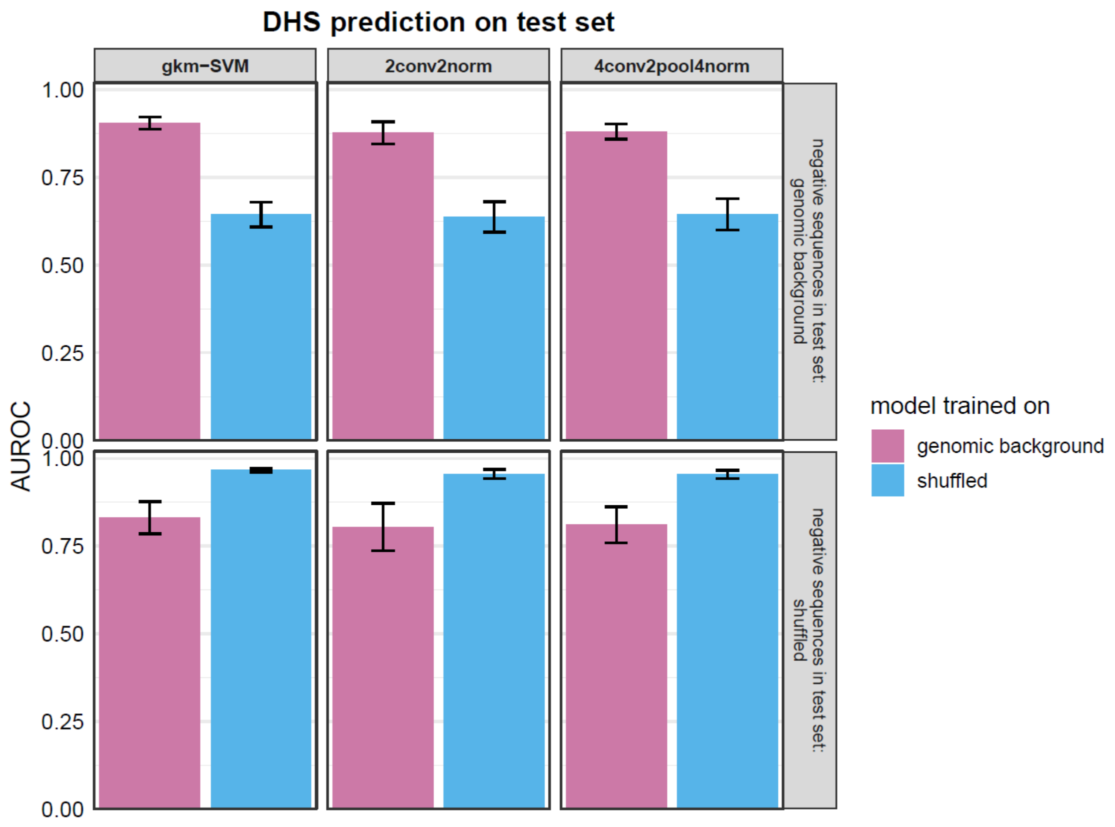

P 14 lines 338 to 348 and Figure S10A: In my mind, this figure contains a key message and one could think about moving it into the main figures. It should be indicated which blue curve belongs to which k-mer and a number/percentage of how many 8-mers were excluded (because they weren't present in the genome) should be given.

We appreciate your suggestions on Figure S10A and changed lines and legends in the figure accordingly. We feel that the qualitative statements and results of statistical tests in the main text are sufficient and decided to keep the figure in the supplement. Regarding the number of excluded kmers, we checked again and noticed that all 65,536 possible 8mers are present in the reference sequence. Thus, no 8mers were removed in these analyses. We adjusted the legend and method descriptions correspondingly.

P16 line 431: While surely obvious to the authors, I initially found it confusing that the model from the previous publication performed so much better in the experimental activity prediction. It would probably help the reader to very briefly recap that the model from publication 23 was trained on a corresponding set of ChIPped regions rather than DHS sites.

We added a sentence to recap the prior analysis in Inoue et al.:

"Inoue et al. [25] analyzed how their enhancer activity readouts correlated with a gkm-SVM model trained from more than 200,000 ENCODE ChIP-seq peaks observed in HepG2 [17]. Our HepG2 models based on DHS sites did not achieve the performance of a Spearman's ρ of 0.28 reported before [25] (see Fig 3)."

Further, we extended the description in Figure 4 to make clear that the model of the original publication was trained on ChIP-seq data and not on DHS sites.

"The dashed black line (Spearman's ρ=0.276) represents a reference value achieved previously by a gkm-SVM using ENCODE ChIP-seq peaks versus matched control sequences [25]."
