# Supplementary material for: The impact of different negative training data on regulatory sequence predictions
Source: PLoS One. 2020 Dec 1;15(12):e0237412. doi: 10.1371/journal.pone.0237412 (PMC7707526; doi:10.1371/journal.pone.0237412)
Supplement: Attachment — Submitted filename: reviewer_responses.pdf [file pone.0237412.s027.pdf]

Comments by the editor:

Specifically, given the amount of discussion triggered by the reviewers' comments on the possible impact of not distinguishing between promoters and enhancers, we feel that it would be important for the authors to more extensively present their rationale and views on this matter (during results interpretation and/or in the discussion section). You may also choose to address additional comments from the reviewers.

We highly appreciated the discussion, but it is still uncommon to apply Deep Learning/open chromatin modeling studies on DHS sites split between promoter-associated and potentially enhancer-associated sites as proposed by the reviewers. There are multiple studies (e.g. SegWay, ChromHMM, ENCODE SCREEN) where authors infer different types of elements from DHS sites and other biochemical readouts, but this is different from predicting element or base-pair level activities. As also agreed by the reviewers, our study did not look into how DHS-derived models should be built in general, but focuses on the very specific question of how the negative set should be chosen when a supervised learning method is applied. We believe that the Discussion is the right section for pointing out that promoter DHS sites have different characteristics than distal DHS sites and that a naïve joined modeling might have its limitations. We have added the following new paragraph at the end of the Discussion:

We note the GC content might only be one aspect where DHS sites marry multiple functional units, with potentially different characteristics. The bimodality apparent in the GC content of the DHS sites used (S17 Figure) is likely due to CpG island/GC-rich promoters, as others have previously described unimodal distributions for enhancers [Lecellier CH et al. Genetics 2018]. This not only argues for good empirical matching of GC characteristics (incl. a long tailed GC distribution of enhancers [Lecellier CH et al. Genetics 2018]), but also prompts an argument that DHS datasets could potentially be better modeled if split-up. It has long been described that enhancers and promoters differ in their histone code [Heintzman ND et al. Nature Genetics 2007] and that they at least partially interact with different DNA binding proteins [Nguyen TA et al. Genome Research 2016, Partridge EC et al. Nature 2020]. However, the lines are blurred and they are overall similar in DNA sequence, chromatin and TF architecture [Andersson R et al. Trends in Genetics 2015; Andersson R & Sandelin A Nature Reviews Genetics 2020]. Further, when starting to define classes, subclasses within promoters immediately come into focus. For example, CpG-rich promoters are associated with ubiquitously expressed genes or complex expression patterns (e.g. during embryonic development) [Mikkelsen TS et al. Nature 2007, Mendenhall EM et al. PLoS Genetics 2010], while CpG-poor promoters are associated with tissue-specific expression [Mikkelsen TS et al. Nature 2007] and similar to enhancers in terms of recruited TFs [Andersson R et al. Nature 2014]. Others have described intragenic enhancers acting as alternative promoters [Kowalczyk MS et al. Molecular Cell 2012] and promoters acting as enhancers of other promoters [Dao LTM et al. Nature Genetics 2017]. This makes a distinction context-dependent [Andersson R et al. Trends in Genetics 2015], something that is not modeled in the CNNs we explored. Even though not explicitly tested in our study, we do not expect genomic background sequences to work better for one type of regulatory sequence but not the other. We identified learners including rare motifs from artificial sequence sets as the main disadvantage of shuffled sequences. As this is linked to the rareness of motifs in the background, this observation is expected to hold for subsets of open chromatin regions. Further, across datasets used here, promoters contribute on average 12% of DHS sites, with GC-rich promoters contributing an even smaller fraction of that. Splitting up DHS peaks into different types of regulatory elements will considerably reduce training data set size, which we identified as a major bottleneck in training (Fig. 4). Together with the biochemical and functional overlaps between these classes, rather than splitting the data, combined Deep Learning models with shared as well as separate layers might be worthwhile to explore in future studies.

Even though we were not required by the editor to address these second round comments, we provide responses to both reviewer's comments below.

Reviewer #1: All comments have been addressed

Reviewer #1: The authors addressed majority of the points I raised.

1. Point 3 and 4. We are happy with the changes made by the authors. Nevertheless, the suggested papers to be citated were the minimum and we were expecting the authors to cite those papers we suggested and others.

Across many reviewer requests seen by us over many studies as both reviewers and authors, this reviewer's understanding of the review process seems unusual. The academic review process is supposed to identify shortcomings in how studies are performed and to improve studies prior to publication. It is not meant as a process where reviewers implement their own views or favorite papers in the manuscripts they review. We have made multiple additions in the introduction of our manuscript based on this reviewer's requests, we now kindly decline to cite any more papers regarding chromatin-associated TF binding.

2. Point 11, the authors explained that part, but did not change the text. Readers might have the same question and I think it is important to be clear.

It is very unlikely that other readers will have the same confusion whether this is about model training or model evaluation. This sentence is in a section entitled "Evaluation tasks and model evaluation". The sentence itself refers to "testing" and the previous sentence to "model evaluation". With all due respect, the reviewer simply misread the sentence.

3. Point 18. The authors mentioned that they do not think the suggested reference is appropriate, but did not provide any explanation for that or any alternative paper.

The paper discusses the topic of "information content" in DNA binding motifs, specifically how multi-cellular organisms require longer TF motifs (i.e. more information content) than yeast and bacteria. However, the paper never discusses specific kmer lengths or modeling of TFs using DNNs/gkm-SVMs. It therefore does not inform parameter choice nor does the comparative nature of the manuscript provide a useful angle for interpreting our results.

Reviewer #2: (No Response)

Reviewer #2:

Major Point 1

R: I completely appreciate that the manuscripts main focus is not on creating the best possible model for open chromatin prediction. The fact remains though that it is possible that background and parameter choice has a different influence depending on what it is that one is trying to predict – and open chromatin regions are clearly a mixed bag. While it may be beyond the scope of this study to look at subgroups of open chromatin regions (this could for example have been done on the first task only to shine some light on how much influence this might have), I find it disappointing that the authors chose to not even discuss this question.

We quantified overlaps with regions around transcriptional start sites (-800,+200 bp of all transcripts annotated in ENSEMBL release 101) for the applied DHS datasets. We see on average 11.87% of the DHS sites potentially being of a promoter origin, with different cell-types ranging from 9.0% to 16.1%.

This highlights that promoters are a clear minority class; CpG-island associated ones even more so. Given the results of our study, i.e. learners including rare motifs from artificial sequence sets as the main disadvantage of shuffled sequences and observing data limitations for training DNN models, a discussion of such minority classes seems only confusing for the reader in the results. We therefore decided to discuss "the mixed bag" issue within the context of other studies in a new paragraph of the Discussion, as provided above.

Other points:

I agree with the authors that a correlation between prediction and experimental readout does not have to be linear to be useful – I have no problem with using rank correlation here. But I do agree with Reviewer 1 that the correlation is poor (significance is easily reached here because of the numbers) and that this is not made sufficiently clear in the text. Instead, as I mentioned in my last comment, a lot of emphasis is placed on the minute effects of different parameters on the (very) weak correlation. I am fully aware that enhancer predictions based on sequence alone are generally not great, and that making the best possible prediction is not the aim of this study.

Without repeating our earlier response, it might be worthwhile to mention that these results should always be considered in the context of applying a binary classification model for the quantitative ranking of elements. The reviewers should appreciate, that model training had no information about quantitative readouts of these DHS sites and only used open and non-open chromatin regions for training. We are then using class probabilities/distances to decision boundaries as the quantitative readouts of a binary model. On top of that comes a technical limitation on the quantitative MPRA readouts, probably limiting a maximum possible correlation to its experimental noise/bias with a Spearman correlation of something like 0.82-0.84 (Klein et al. Nature Methods 2020, Suppl. Fig. 5). Previously used linear models using ***quantitative*** inputs (Inoue F et al. Genome Research 2017) achieved a Spearman correlation of 0.52, while gkm-SVM models in the same study achieved 0.28 (and a combination of both of 0.55). gkm-SVM performance in our study is completely on par with the previous results and we show that overcoming training data limitation will bring DNNs to similar or better performance (Fig. 4).

Again, I find it disappointing that the authors missed the opportunity to include more information on the relationship between prediction and experimental readout – for example like the scatterplot of (ranked) prediction vs experimental readout I suggested previously.

Not reiterating our previous response, scatter plots for such low correlations are hard to interpret for the human eye. It is impossible for humans to judge significance and differences between multiple point clouds. For this reason, we added the confidence levels in the bar charts (S14 and S15 Figures) with the revisions. Having different labels for promoters and enhancers in a scatter plot would further complicate the interpretation for a human reader, as we are dealing with uneven class representation (i.e. 1 out 10 for promoter-associated DHS sites among all DHS, as outlined above).

R: Something that I noted when re-reading the manuscript is that at two points in the text relating to Figure 4 it is suggested that gkm-SVM models were trained up to a maximum set size of 350 K, however, the last data point is at 200 K. This should be corrected.

Thank you for spotting this inconsistency, we somehow missed this last data point of 350 K in our figure and now added it to the plot.
